# *Grindelia* *mutabilis* (Asteraceae: Astereae), a New South American Species and a Link for Synonymizing *Notopappus*

**DOI:** 10.3390/plants15050760

**Published:** 2026-03-01

**Authors:** Fernando Fernandes, Bruno de Souza, João Iganci, Tatiana Teixeira de Souza-Chies, Gustavo Heiden

**Affiliations:** 1 Programa de Pós-Graduação em Botânica, Universidade Federal do Rio Grande do Sul, Porto Alegre 91501-970, RS, Brazil; joaoiganci@gmail.com (J.I.); tatiana.chies@ufrgs.br (T.T.d.S.-C.); gustavo.heiden@embrapa.br (G.H.); 2 Departamento de Botânica, Universidade Federal do Rio Grande do Sul, Porto Alegre 91501-970, RS, Brazil; brunodesouza@ufrgs.br; 3 Departamento de Botânica, Universidade Federal de Pelotas, Campus Capão do Leão, Pelotas 96010-900, RS, Brazil; 4 Embrapa Clima Temperado, Rodovia BR 392, km 78, Caixa Postal 403, Pelotas 96010-971, RS, Brazil

**Keywords:** *Asteroideae*, *Compositae*, endangered species, endemism, grassland, *Machaerantherinae*, savanna, taxonomy

## Abstract

*Grindelia mutabilis* (Asteraceae, Astereae), a new species from Brazil endemic to the Espinal Ecoregion of the Río de La Plata Grasslands Bioregion and Pampa Province of the Chaco Biogeographical Domain, is proposed and illustrated. The new species is characterized by a combination of traits: small, rosette cespitose habit, linear to linear–oblanceolate leaves, light-yellow to pastel salmon ray florets, three-winged ray floret cypselae bearing a pappus of two to four elements and two-winged disc floret cypselae bearing a pappus of two elements. It has a highly restricted habitat and is known exclusively within Parque Estadual do Espinilho in Rio Grande do Sul, Brazil. Preliminary conservation assessments classify the new species as Critically Endangered. We provide illustrations and photographs, as well as a distribution map with an identification key for the South American *Grindelia* species with winged cypselae. The intriguing morphology of this species combines characters traditionally regarded as diagnostic for *Notopappus*, a genus segregated from *Haplopappus* and *Grindelia*. Previously published phylogenetic studies of related taxa indicate that the recognition of *Notopappus* as monophyletic is not supported and render *Grindelia* as non-monophyletic too. Based on this combined morphological evidence and existing phylogenetic hypotheses, we reaffirm the non-monophyly of *Notopappus* and formally propose its synonymization under *Grindelia* s.l.

## 1. Introduction

*Grindelia* Willd. (Asteraceae) comprises 72 species, with most species being xerophytes occurring in a disjunct distribution between the temperate regions of North and South America [1,2,3,4,5,6,7,8]. Regarding the relationships of *Grindelia* to other taxa in the subtribe Machaerantherinae [9], previous phylogenies [10,11] have shown that the genus is sister to a clade comprising North American genera *Isocoma* Nutt., *Rayjacksonia* R.L.Hartm. & M.A.Lane, and *Xanthocephalum* Willd. North American genera *Hazardia* Greene, *Pyrrocoma* Hook., and *Lessingia* Cham., as well as the South American *Haplopappus* Cass., were recovered as a polytomy with the clade composed of *Isocoma*, *Rayjacksonia*, and *Xanthocephalum* in the nrDNA trees but were placed in a separate clade in the cpDNA restriction-site phylogenetic reconstruction [11].

Recent phylogenetic studies, such as the Plant and Fungal Tree of Life (PAFTOL) initiative from Kew [12,13], based on the Angiosperm353 probe kit, confirm *Isocoma* as sister to *Grindelia*, with both closely related to *Pyrrocoma*. These genera are, in turn, sister lineages to a clade formed by *Corethrogyne* DC. and *Haplopappus*. However, these more recent studies did not sample all genera previously considered in phylogenetic analyses of the subtribe, leaving some taxonomic hypotheses untested.

Within the Astereae tribe, the Machaerantherinae subtribe is traditionally represented in South America by only two genera: *Grindelia*, with 31 species mostly distributed across the Southern Cone and the Andes, reaching as far north as Bolivia and Peru, and *Haplopappus*, with 75 species and a similar distribution, although extending further north up to Ecuador [1,14,15]. This long-standing generic framework was altered by the proposal of *Notopappus* Klingenberg, a genus segregated from *Grindelia* and *Haplopappus* based on five South American species, occurring in South America in northwestern and southern Argentina and in central and southern Chile [16].

Despite its recognition in some subsequent treatments [16], the circumscription of *Notopappus* has already been the subject of criticism regarding its taxonomic delimitation [17], since the characters used to justify its segregation were not evaluated in light of the full range of morphological variation observed in *Grindelia*. In addition, phylogenetic analyses of *Grindelia* indicate that the species assigned to *Notopappus* may be nested within *Grindelia* rather than constituting a distinct generic lineage [8], reinforcing the need to reassess its generic limits.

In Brazil, Machaerantherinae is represented solely by *Grindelia*, which reaches one of the geographical limits of its distribution there, with seven species occurring primarily in the Chacoan domain and along its boundaries with the Paraná domain [6]. Within this region, species are mostly associated with coastal sandy plains and rocky outcrops. Two species, *G. atlantica* Deble & A.S.Oliveira and *G. gaucha* Deble & A.S.Oliveira, are endemic to Brazilian Pampean environments [3]. In addition, *G. rheophila* Fern.Fern. & G.Heiden is endemic to the ecotonal region between the Pampean grasslands and the Paraná forest provinces in Brazil [7]. *G. buphthalmoides* DC., *G. pulchella* Dunal, *G. puberula* Hook. & Arn. and *G. scorzonerifolia* Hook. & Arn. have partially overlapping distributions extending into Argentina and Uruguay [1,6].

The Espinal province extends as an irregular arch from the borderlands of Brazil and Uruguay with central Corrientes and north of the Entre Ríos provinces in Argentina, south to Buenos Aires. The dominant vegetation is a seasonally dry forest interspersed by palm groves, grasslands and halophilous steppes associated with a strong edaphic character along geographic depressions and riverbanks [18]. Despite the distinct physiognomy, this province was later synonymized with the Pampean province [19] based on biogeographic analysis. Even so, this area is still considered part of the Chacoan domain. Besides these phytogeographic reinterpretations, the region is recognized by the World Wide Fund for Nature (WWF) [20] as the Espinal ecoregion, which coincides with the Ñandubay district [18], and belongs to the Río de la Plata grasslands biogeographic region [20].

Floristically, Ñandubay vegetation is dominated by four tree species growing under semi-arid conditions, interspersed with grasslands: espinilho (*Vachellia caven* (Molina) Seigler & Ebinger), inhanduvá (*Neltuma affinis* (Spreng.) C.E.Hughes & G.P.Lewis), algarrobo (*Neltuma nigra* (Griseb.) C.E.Hughes & G.P.Lewis), and quebracho-branco (*Aspidosperma quebracho-blanco* Schltdl.) [21]. In Brazil, this vegetation previously occupied more extensive areas in southern Rio Grande do Sul state than today, where it is currently extremely restricted and remains poorly protected only in the Parque Estadual do Espinilho (Espinilho State Park). This region features a climate without a defined dry season but is subject to the desiccating effects of cold fronts, favoring the development of ecosystems characterized by savanna formation [22]. This conservation unit is in the municipality of Barra do Quaraí, where it stands out as a unique ecological area in the country. Situated at the confluence of the Quaraí and Uruguay rivers, the park lies within the Río de la Plata basin and has been historically associated with the Espinal province of the Chaco domain.

During herbarium studies and fieldwork conducted as part of a taxonomic revision of *Grindelia* in Brazil, a distinct new species from Parque Estadual do Espinilho was identified. These populations had previously been referred to as *Grindelia scorzonerifolia*, but detailed comparative analyses revealed a unique combination of characters that falls within the morphological variation of *Grindelia* and does not support their placement in *Notopappus* or the recognition of *Notopappus* as a distinct genus. This finding highlights the inadequacy of the characters previously used to segregate *Notopappus* and reinforces the need for a reassessment of generic limits within South American Machaerantherinae. Accordingly, this study proposes a new species and presents the morphological and taxonomic evidence supporting the synonymization of *Notopappus* under *Grindelia*.

## 2. Results

### 2.1. Taxonomic Treatment

**Figure 1:**
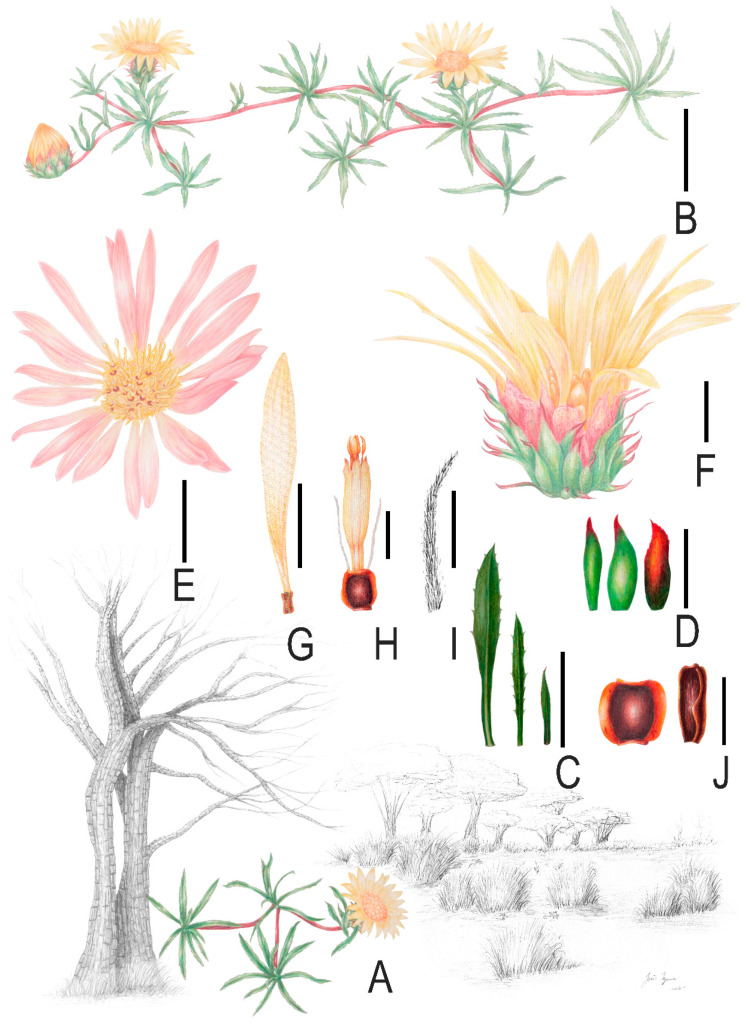
*Grindelia mutabilis* (Asteraceae). (**A**) Habitat. (**B**) Cespitose rosette habit with spreading branches; scale bar = 0.3 m. (**C**) Leaves and bracts, from left to right: leaf, bract distant from the capitulum, and bract closest to the capitulum; scale bar = 20 mm. (**D**) Phyllaries, from left to right: outer, middle, and inner phyllaries; scale bar = 4 mm. (**E**) Frontal view of the capitulum showing ray florets with pastel salmon-colored ligules; scale bar = 10 mm. (**F**) Lateral view of the capitulum showing ray florets with light-yellow ligules; scale bar = 10 mm. (**G**) Ray floret; scale bar = 5 mm. (**H**) Disc floret; scale bar = 2 mm. (**I**) Pappus; scale bar = 2 mm. (**J**) Cypselae, from left to right: cypsela of a disc floret and cypsela of a ray floret; scale bar = 1 mm. Illustration by João Iganci.

**Figure 2:**
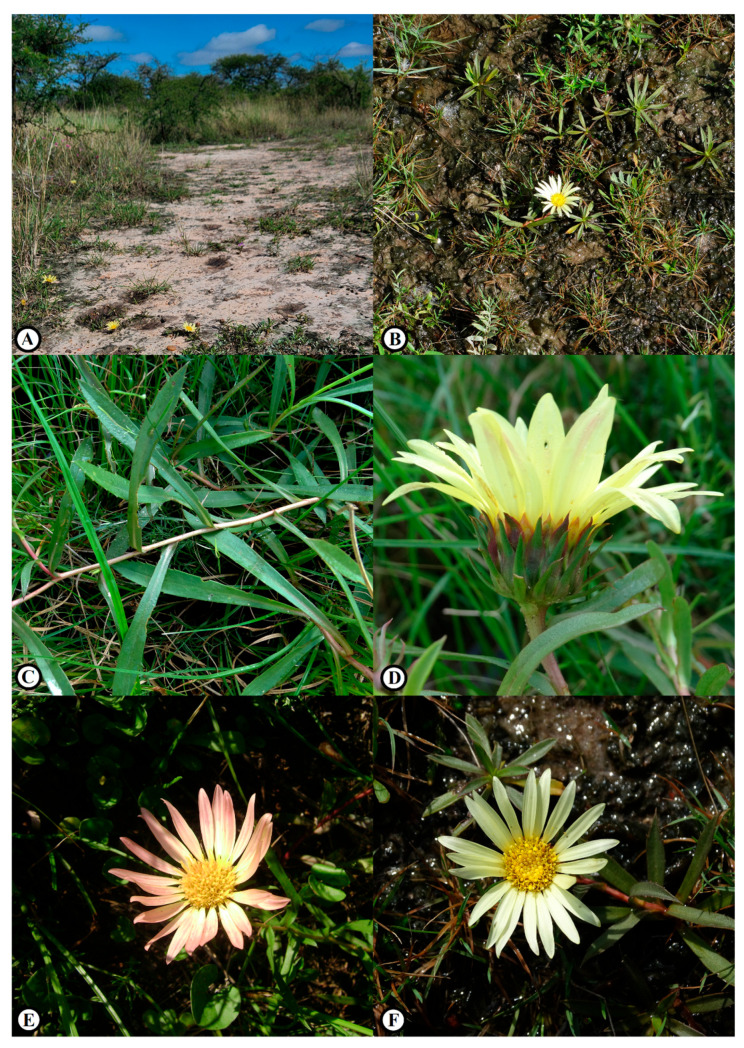
*Grindelia mutabilis* (Asteraceae). (**A**) Habitat on sandy, halophilous soils within the Espinal/Ñandubay savanna of Espinilho State Park, Barra do Quaraí, Rio Grande do Sul, Brazil. (**B**) Cespitose rosette habit with spreading branches. (**C**) Leaves. (**D**) Lateral view of the capitulum. (**E**) Capitulum with pastel-salmon ray florets. (**F**) Capitulum with light-yellow ray florets. Pictures by F. Fernandes (**A**), B. de Souza (**B**–**D**,**F**), and M. Grings (**E**).

***Grindelia mutabilis*** Fern.Fern. & G.Heiden, ***sp. nov.*** (Figure 1 and Figure 2)

**Type:** Brazil, Rio Grande do Sul: Barra do Quaraí, Parque Estadual do Espinilho, 30°11′22.5″ S, 57°29′46.7″ W, 51 m a.s.l., 16 Dec. 2009, *M. Grings & R. Paniz 983* (holotype: ICN 163240!, isotype PACA 109075!).

#### 2.1.1. Diagnosis

*Grindelia mutabilis* differs from all other species in the genus by the unique combination of cespitose rosette habit 0.2–0.3 m tall; leaves linear to linear–oblanceolate; ray corollas light yellow to pastel salmon; three-winged ray floret cypselae bearing a pappus of two to four elements and two-winged disc floret cypselae bearing a pappus of two elements.

#### 2.1.2. Etymology

The specific epithet *mutabilis* (Latin for “changeable” or “variable”) refers to the ligules of the ray florets that change from light yellow to pastel salmon along the maturity.

#### 2.1.3. Description

Low-growing, prostrate **shrubs** with a cespitose rosette habit and spreading branches, 0.2–0.3 m tall, sympodial. **Stems**: prostrate, cylindrical, bark light brown to green, smooth, slightly striate; sparsely pubescent, glabrate with maturity, glandular and rarely non-glandular normal trichomes; internodes in upper vegetative and flowering branches 1–15 mm long. **Leaves:** 45–53 × 4–5 mm, alternate, light green, concolorous, sessile, entire; blade chartaceous, linear to linear–oblanceolate; base tapering, semi-amplexicaule; apex acute; margin serrate to sparsely serrate, occasionally entire at the base and sparsely serrate distally, with uncinate teeth, not revolute; venation pinnate; indumentum: sparsely pubescent, glandular with sparse normal trichomes. **Bracts:** 4.4 × 4.6 mm, alternate, light green, concolorous, sessile, linear to linear–oblanceolate; blade chartaceous, entire; base tapering, semi-amplexicaule; apex acute; margin serrate to sparsely serrate, occasionally entire at the base and sparsely serrate distally, with uncinate teeth, not revolute; venation pinnate; indumentum: sparsely pubescent, glandular with sparse normal trichomes. **Bracteoles**: 9–53 × 1–4 mm, alternate, light green, concolorous, sessile, linear to linear–oblanceolate, as they ascend, the bracteoles become progressively smaller and more similar in appearance to the phyllaries; blade chartaceous, entire; base tapering, semi-amplexicaule, gradually decreasing toward the capitulum; apex acute; margin entire to serrate, sometimes sparsely serrate or entire at the base and sparsely serrate distally, with uncinate teeth, not revolute; venation pinnate; indumentum: sparsely pubescent, glandular with sparse normal trichomes. **Capitulum:** solitary; involucre 7–10 × 13–19 mm, 5–7-seriate. **Phyllaries:** 38–45; outer phyllaries 4.8–6 × 1.1–2 mm, light green with vinaceous tones on the edges, lanceolate, oblong to elliptical, almost coriaceous; base decurrent, adpressed; midportion patent; apex long attenuated, supinate; margin regular; indumentum: sparsely pubescent with glandular trichomes. Second series 6–7 × 1.7–2 mm, light green with vinaceous tones on the edges, lanceolate, oblong to elliptical almost coriaceous; base decurrent, adpressed; midportion patent; apex long attenuated, supinate; margin regular; indumentum: sparsely pubescent with glandular trichomes. Intermediate series 6.5–8 × 2.0–2.5 mm, light green with stramineous margins, oblong to elliptical, almost coriaceous to papyraceous and stramineous at the margins; base decurrent, adpressed; midportion patent to straight; apex attenuated; margin smooth; indumentum: sparsely pubescent with glandular trichomes. Inner phyllaries 7–7.5 × 1.6–2.2 mm, light green to stramineous, oblong to elliptical, papyraceous to stramineous at the margins; base decurrent, adpressed; apex attenuated, supinate; margin smooth; indumentum: sparsely pubescent with glandular trichomes. **Receptacle**: flat. **Ray florets**: 18–27, pistillate, corolla light yellow to pastel salmon; tube 3.0–3.2 × 0.5–0.7 mm, sparsely pubescent with glandular trichomes; throat puberulous with glandular trichomes; limb 10–12 × 2–2.2 mm elliptical to oblong; apex acute; indumentum puberulent to glabrous, trichomes glandular when present. Ovary 1–1.3 × 0.4–0.5 mm, ovoid; indumentum puberulent to glabrous, trichomes glandular when present; style 4–5 mm long; style arms 1–1.1 mm long, apex acute, sparsely pubescent. **Disc florets:** 63–64, perfect; corolla tubular 4.7–5.3 × 0.4–0.9 mm yellow; tube sparsely pubescent with glandular trichomes; throat puberulous with glandular trichomes; lobes puberulent to glabrous, trichomes glandular when present. Anthers 2.8–3 mm long, light yellow. Ovary 0.7–1 × 0.5–0.7 mm, ovoid; indumentum: sparsely pubescent to glabrous, trichomes glandular when present; style 4–6 mm long; style arms 1–1.2 mm long, apex acute, sparsely pubescent. **Ray cypselae:** 1–1.8 × 0.3–0.7 mm, ovate-cylindrical, laterally compressed, dark brown, glabrous; apex truncate, tri-winged 0.1–0.2 mm, light brown. Pappus 3.1–4 mm long, dorsiventrally flattened, and ciliate along the margins, 2 (3–4) pappus elements. **Disc cypselae:** 1–1.1 × 0.5–0.7 mm, ovate, laterally compressed, dark brown, glabrous; apex truncate, bi-winged 0.1–0.3 mm, light brown; Pappus 3.1–4 mm long, paleaceous, and ciliate along the margins; two pappus elements, deciduous.

#### 2.1.4. Paratypes

BRAZIL, Rio Grande do Sul: Barra do Quaraí, Parque Estadual do Espinilho, 12 Oct. 2017, M. Köhler, J. Külkamp, C.S. Rabuske & M.V.B. Soares 225 (ICN 198551!); Uruguaiana [Barra do Quaraí], Parque Estadual do Espinilho, 17 Nov. 1984, M. Sobral 3426 (ICN 065419!; F2007256, digital image!); Barra do Quaraí, Parque Estadual do Espinilho, 25 Nov. 2021, P.J.S. Silva Filho & G. Fockink 2450 (SMDB 21942, digital image!); Barra do Quaraí, Parque Estadual do Espinilho, 26 Nov. 2021, P.J.S. Silva Filho & G. Fockink 2475 (SMDB 21967, digital image!). Barra do Quaraí to Uruguaiana, railroad track, 15 Jan. 1941, B. Rambo s.n. (PACA4492!).

### 2.2. Phenology

*Grindelia mutabilis* was recorded flowering and fruiting from October to January.

### 2.3. Distribution and Ecology

**Figure 3:**
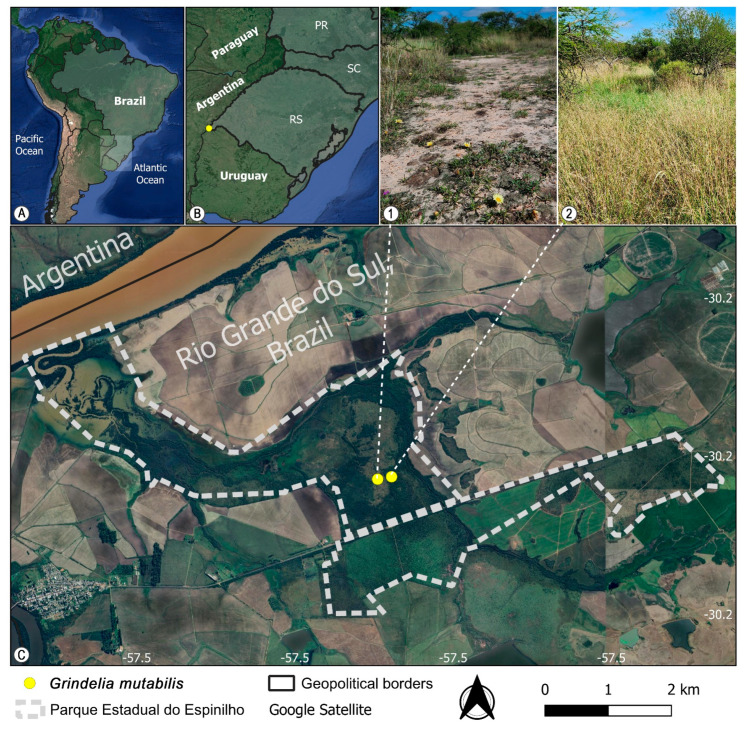
Geographic context and distribution of *Grindelia mutabilis* (Asteraceae: Astereae). (**A**) South America. (**B**) Rio Grande do Sul state, southern Brazil. (**C**) Parque Estadual do Espinilho, municipality of Barra do Quaraí. (**1**) Location where the new species was recorded during recent field expeditions; area with sparse vegetation and rocky–sandy soil. (**2**) Type specimen locality; currently characterized by dense vegetation, where the species is no longer found.

*Grindelia mutabilis* was found growing on sandy soils along the herbaceous layer of Espinal/Ñandubay savanna vegetation (Espinilho State Park, Barra do Quaraí, Rio Grande do Sul, Brazil), where it integrates the grassy–steppe component associated with open areas (Figure 3). This environment is characterized by a mosaic of xerophytic vegetation interspersed with seasonally dry grasslands and seasonally dry forests that, unfortunately, represents one of the last well conserved remnants of the Espinal ecoregion in Brazil. Despite its occurrence close to the triple border with Argentina and Uruguay, the species has, so far, not been found in the neighboring countries in herbaria or fieldwork. A historical collection made in 1941 (85 years ago; PACA4492) records the species from a former railway line between Barra do Quaraí and Uruguaiana; however, because this railway no longer exists and the area has been heavily anthropized, the exact locality cannot be verified, nor can the current persistence of the species in the area be confirmed [23].

### 2.4. Preliminary Conservation Status

*Grindelia mutabilis* is classified as Critically Endangered (CR) following the IUCN (International Union for Conservation of Nature) [24] Red List assessment criteria: B1ab(ii,iii,iv)c(ii,iii)+2ab(ii,iii,iv)c(ii,iii). This classification is based on the species’ extremely limited geographic distribution, with an Extent of Occurrence (EOO) of 4 km^2^ and an Area of Occupancy (AOO) estimated at 762 m^2^, calculated from the actual area where individuals were observed. These spatial metrics were obtained through analysis using GeoCAT (Geospatial Conservation Assessment Tool) [25] and direct field measurement, which showed that the species occurs in only two nearby localities. All applicable IUCN criteria (A–D) were evaluated; criterion E was not applied due to an insufficient amount of data for quantitative extinction risk analysis.

*Grindelia mutabilis* is, so far, known from a single population of the type locality. Although this area is legally protected, the population is extremely small, comprising only ca. 35 mature individuals, and is restricted to a narrow edaphic niche. Independent ecological evidence indicates that this species is an indicator of blanqueales, a type of halomorphic vegetation characterized by alkaline and saline soils and naturally open vegetation [26]. In the type locality (Figure 3(2)), *G. mutabilis* was not relocated during recent fieldwork, likely because of vegetation densification following habitat alteration, which reduces the availability of open microsites required by the species. However, individuals persist at a nearby site approximately 220 m away (Figure 3(1)), where rocky and sandy substrates maintain open conditions and limit competitive exclusion. Despite being recorded at two points, these occurrences are interpreted as a single population due to habitat continuity and the absence of physical or ecological barriers to gene flow.

Although the species shows a strong dependence on edaphically extreme microhabitats, its rarity cannot be explained solely by habitat specialization. Land-use and land-cover data derived from satellite imagery clearly document extensive and long-term conversion of savanna ecosystems in the region [27], historically driven by rice cultivation and, more recently, intensified by the expansion of soybean monocultures. This process has drastically reduced the extent of suitable habitats. In addition, cattle grazing has promoted the densification of the herbaceous shrub layer, a pattern commonly associated with selective herbivory that favors unpalatable and structurally more rigid species. Field observations further indicate widespread invasion by exotic grass *Eragrostis plana* Nees (Love grass or Annoni grass) in the surroundings and within the protected area, likely facilitated by livestock management dispersal, representing a significant additional threat to native vegetation. Taken together, habitat loss, grazing-induced structural changes, and biological invasion highlight the urgency of targeted population monitoring and the implementation of both in situ and ex situ conservation actions.

## 3. Discussion

### 3.1. Grindelia mutabilis and the Allied South American Species with Winged Cypselae

This discussion includes *Grindelia mutabilis* and other South American species presenting truly winged cypselae, e.g., those that have been transferred to the *Notopappus* genus, as well as related species exhibiting marginal structures that may resemble wings, such as keels or slight projections. These taxa are considered here based on the examination of herbarium specimens and the compilation of descriptions and illustrations [1,15,28]. They share morphological similarities, especially in cypsela shape and reduction in the number of pappus elements, which can lead to misidentification. At the end, an identification key is provided with the aim of assisting in the accurate delimitation of *G. mutabilis* from morphologically similar species.

The species described here as *Grindelia mutabilis* had previously been identified as *G. scorzonerifolia*. However, detailed morphological analyses clearly distinguish the new species from *G. scorzonerifolia*, which differs by its shrubby habit with woody, prostrate stems and ascending herbaceous shoots (vs. low-growing, prostrate shrubs with a cespitose rosette habit andspreading branches in *G. mutabilis*); petiolate, narrowly elliptic, lobed, pinnatifid or pinnatisect leaves (vs. entire, sessile, linear to linear–oblanceolate leaves); yellow (vs. light-yellow to pastel-salmon) ray corollas; prismatic cypselae, even in disc florets (vs. ovate cypselae in the new species); and a pappus composed of four to five awns (vs. two (three to four) in *G. mutabilis*).

*Grindelia mutabilis* is placed within *Grindelia* based on a suite of diagnostic characters consistent with the genus [1,14,28]. The new species is assigned to *Grindelia* because it exhibits all the diagnostic traits of the genus, including capitula with campanulate involucres, bracts arranged in multiple series (5–7) with sclerified bases, thick-walled cypselae, and a pappus composed of few deciduous elements (2(3–4)). The presence of winged cypselae, while consistent with the variation observed in *Grindelia*, is an uncommon trait restricted to a few South American taxa and one in North America (*G. tricuspis* (Sch.Bip.) Adr.Bartoli & Tortosa) [1,4]. Among the 72 species currently recognized in the genus, only eleven taxa exhibit this feature. This character was one of the main traits used to justify the segregation of *Notopappus* as a distinct genus [15].

*Grindelia mutabilis* differs from most South American species by its pappus, which consists of two (three to four) elements in the ray florets and only two in the disc florets and by its three-winged cypselae in ray florets and two-winged cypselae in disc florets. Three other taxa in *Grindelia* possess well-defined winged cypselae and a reduced number of pappus elements, which may lead to confusion with the new species. *Grindelia cabrerae* Ariza var. *alatocarpa* Ariza bears winged fruits and two to four (five) pappus elements—features shared with the new species. However, the variety is an erect shrub with elliptic leaves, whereas the new species is a prostrate shrub with linear to linear–oblanceolate leaves. Additionally, *G. mutabilis* has significantly broader phyllaries in comparison with the variety: outer phyllaries of 1.1–2 mm vs. 0.5–0.8 mm and inner phyllaries of 1.6–2.2 mm vs. 1–1.5 mm. The ray-floret corolla also differs: the new species has a light-yellow to pastel-salmon corolla, while the variety exhibits a bright yellow one.

*Grindelia chiloensis* (Cornel.) Cabrera, which also has ovate and winged cypselae, is an erect shrub reaching up to 1 m in height. It bears elliptic to narrow elliptic leaves, whereas *G. mutabilis* is a prostrate shrub up to 30 cm tall with linear to linear–oblanceolate leaves. *G. chiloensis* also differs by having 6–13 pappus elements (vs. 2(3–4) in *G. mutabilis*).

*Grindelia coronensis* A.Bartoli & Tortosa, which also has winged cypselae, is easily distinguished from the new species by its petiolate, spatulate leaves with an obovate blade (vs. sessile, linear to linear–oblanceolate leaves in *G. mutabilis*). In addition, *G. coronensis* has five to six pappus elements (vs. two (three to four) in *G. mutabilis*).

In addition to these three taxa, other South American *Grindelia* were later transferred to *Notopappus* [15] and are also compared to *G. mutabilis*. Although their original descriptions did not mention the presence of winged cypselae, this character was among the key features used [15] to justify the segregation of *Notopappus* as a distinct genus. These taxa include *Grindelia andina* Phil. [=*Notopappus andinus* (Phil.) Klingenb.], *Grindelia prunelloides* (Poepp.) A.Bartoli & Tortosa [=*Notopappus prunelloides* (Poepp. ex Less.) Klingenb.], *Grindelia prunelloides* var. *discoides* Tortosa & Adr.Bartoli [=*Notopappus chryseus* (Kuntze) Klingenb.], and *Grindelia anethifolia* (Phil.) Adr.Bartoli & Tortosa [=*Notopappus pectinatus* (Phil.) Klingenb.]. They can be distinguished from *G. mutabilis* based on a combination of morphological characters, as detailed below.

*Grindelia andina* is distinguished from *G. mutabilis* by its erect stems reaching 30 cm or more in height (vs. 20–30 cm tall rosette cespitose in *G. mutabilis*). The leaves of *G. andina* are lanceolate, coarsely serrate, and up to 40 mm long × 10 mm wide (vs. linear to linear–oblanceolate, 45–53 × 4–5 mm, serrate to sparsely serrate in *G. mutabilis*). This species also has tetragonal, glabrous cypselae with a pappus of serrated hairs about 6 mm long, fused at the base, forming a basal ring (vs. *G. mutabilis* with laterally compressed, tri- and bi-winged cypselae and a pappus of two (three to four) deciduous elements, 3.1–4 mm long, lacking a basal ring).

*Grindelia anethifolia* is distinguished from *G. mutabilis* by its erect, branched stems reaching 0.5–1 m in height (vs. a 20–30 cm tall rosette cespitose in *G. mutabilis*). Its leaves are elliptic, pinnatifid, and 22–45 × 14–20 mm, with sclerotized teeth and sessile glandular trichomes on both surfaces (vs. linear to linear–oblanceolate leaves, 45–53 × 4–5 mm, entire to serrate, with sparse glandular indumentum in *G. mutabilis*). Cypselae in *G. anethifolia* are tri- or tetra-angular, with 23–58 2–8 mm long scabrous, cylindrical pappus elements united by a basal ring (vs. laterally compressed, tri- and bi-winged cypselae in *G. mutabilis*, with two (three to four) 3.1–4 mm long dorsiventrally flattened, deciduous pappus elements, lacking a basal ring).

*Grindelia prunelloides* is distinguished from *G. mutabilis* by its cushion-forming, rhizomatous habit and very short stature of 3–15 cm tall (vs. 20–30 cm tall rosette cespitose, non-rhizomatous shrubs in *G. mutabilis*). The leaves of *G. prunelloides* are spatulate, 11–40 × 3–6 mm, long–petiolate, with crenate to pinnatilobate margins and an obtuse apex (vs. linear to linear–oblanceolate, 45–53 × 4–5 mm, sessile, serrate to sparsely serrate, with an acute apex in *G. mutabilis*). The cypselae of *G. prunelloides* are prismatic and 2.5–4 mm long, and the pappus consists of 35–80 0.5–9 mm long unequal capillary bristles (vs. laterally compressed, tri-winged cypselae in the ray florets and bi-winged in the disc florets of *G. mutabilis*, with two (three to four) 3.1–4 mm long dorsiventrally flattened, ciliate pappus elements). *Grindelia prunelloides* var. *discoides* Tortosa & A.Bartoli is distinguished from *G. prunelloides* by the absence of ray florets in its capitula.

Certain species exhibit structures that can be confused with wings or keels and are herein distinguished from *G. mutabilis* as follows:

*Grindelia covasii* Adr.Bartoli & Tortosa differs from *G. mutabilis* by its erect habit reaching 50–100 cm tall (vs. 20–30 cm tall rosette cespitose shrubs in *G. mutabilis*). The leaves of *G. covasii* are sessile, narrowly obovate, and larger (38–67 × 15–27 mm), with an obtuse apex and irregularly dentate margins with sclerotized awns (vs. linear to linear–oblanceolate, 45–53 × 4–5 mm, acute apex, serrate to sparsely serrate margins in *G. mutabilis*). Cypselae are obovoid, compressed, and 4–5 mm long, with a pappus of 5–13 flattened, sparsely ciliate awns (vs. laterally compressed, tri-winged ray cypselae and bi-winged disc cypselae in *G. mutabilis*, with two (three to four) 3.1–4 mm long dorsiventrally flattened, deciduous pappus elements, lacking a basal ring).

*Grindelia patagonica* Adr.Bartoli & Tortosa differs from *G. mutabilis* by its erect stems reaching 30–50 cm tall (vs. 20–30 cm tall rosette cespitose, sparsely pubescent stems in *G. mutabilis*). The leaves of *G. patagonica* are sessile, narrowly obovate, and 23–42 × 6–12 mm, with an obtuse apex and irregularly dentate margins (vs. linear to linear–oblanceolate, 45–53 × 4–5 mm, acute apex, serrate to sparsely serrate margins in *G. mutabilis*). The capitula in *G. patagonica* is larger, i.e., 33–40 mm in diameter, with 7–12 unequal series of involucral bracts (vs. solitary, 7–10 × 13–19 mm, 5–7 series in *G. mutabilis*). The cypselae of *G. patagonica* are narrowly ovoid, flattened, and about 3 mm long, with a pappus of 6–10 2.5–5 mm long, unequal paleaceous, sometimes twisted, ciliate bristles (vs. laterally compressed, tri-winged ray cypselae and bi-winged disc cypselae in *G. mutabilis*, with two (three to four), 3.1–4 mm long, dorsiventrally flattened, deciduous pappus elements, lacking a basal ring).

Lastly, *Grindelia ventanensis* Adr.Bartoli & Tortosa differs from *G. mutabilis* by its radicant stems (vs. non-rooting stems in *G. mutabilis*). The leaves of *G. ventanensis* are elliptic, larger (28–77 × 8–13 mm), irregularly dentate (vs. linear to linear–oblanceolate, 45–53 × 4–5 mm, serrate to sparsely serrate in *G. mutabilis*). Cypselae are obovoid, flattened, and 7–8 mm long (vs. ovate–cylindrical, laterally compressed, and 1–1.8 × 0.3–0.7 mm in *G. mutabilis*). The pappus consists of four to six capillary awns (vs. two (three to four) dorsiventrally flattened, deciduous pappus elements in *G. mutabilis*).

### 3.2. Key to Grindelia Species in South America with Winged or Laterally Compressed Cypselae

1 Plants with radicant stems … 2
1′ Plants with non-radicant stems … 4
2 Capitula discoid … *G. prunelloides* var. *discoides* (=*N. chryseus*)
2′ Capitula radiate … 3
3 Plants cushion-forming, rhizomatous, 3–15 cm tall; leaves long-petiolate, spatulate, margins crenate-lobed or pinnatifid; capitula discoid or radiate, 15–37 mm diameter; cypselae prism-shaped, 2.5–4 mm long; pappus with 35–80 unequal pappus elements … *G. prunelloides* (=*N. prunelloides*)
3′ Plants prostrate with ascending stems, rooting distally, up to 30 cm tall; leaves sessile with blade narrowing into a pseudo-petiole, elliptical, irregularly dentate; capitula radiate, 40–60 mm diameter; cypselae obovoid, flattened, 7–8 mm long; pappus with 4–6 pappus elements … *G. ventanensis*
4 Leaves linear to linear–oblanceolate; ray corolla light yellow to pastel salmon … *G. mutabilis*
4′ Leaves elliptic, narrowly elliptic, lanceolate, spatulate, obovate, lobed, pinnatifid, or pinnatisect; ray corolla bright yellow … 5
5 Leaves lanceolate; cypselae tetragonal … *G. andina* (=*N. andinus*)
5′ Leaves elliptic, narrowly elliptic, spatulate, obovate, lobed, pinnatifid, or pinnatisect; cypselae compressed, ovoid to ellipsoid, or prismatic … 6
6 Stem prostrate ... *G. scorzonerifolia*
6′ Stem erect ... 7
7 Leaves sessile, elliptic, pinnatifid, lobed almost to midrib; 23–58 pappus elements … *G. anethifolia* (=*N. pectinatus*);
7′ Leaves entire to dentate or serrate, not pinnatifid; 2–13 pappus elements … 8
8 Leaves spatulate with an obovate blade; petioles well developed, 20–30 mm long … *G. coronensis;*
8′ Leaves elliptical or obovate; sessile or with a pseudo-petiole ≤10 mm … 9
9 Leaves elliptical … 10
9′ Leaves obovate to narrowly obovate … 11
10 Stems and phyllaries with stipitate glandular trichomes; leaves 15–30 × 2–6 mm; involucre 5–9 mm tall; cypselae prismatic, 3.0–3.5 mm long; pappus elements 2–4(–5), 4–5 mm long … *G. cabrerae* var. *alatocarpa*
10′ Stems and phyllaries with sessile glandular trichomes; leaves 33–91 × 7–21 mm; involucre 10–18 mm tall; cypselae ovate, 5.5–6 mm long; pappus elements 6–13, 6–13 mm long … *G. chiloensis*
11 Leaves 15–27 mm wide; outer phyllaries elliptic, 10–13 mm long; cypselae obovoid … *G. covasii*
11′ Leaves 6–12 mm wide; outer phyllaries triangular, 4–7 mm long; cypselae narrowly obovoid … *G. patagonica*

### 3.3. Synonymization of Notopappus Under Grindelia

The classification of *Grindelia* has undergone significant changes over recent years, particularly regarding South American species. In addition to the description of new species, taxonomic notes and nomenclatural corrections have been published [28,29,30,31]. The most notable change [15] separated four taxa from the South American species of *Grindelia*, originally described in *Haplopappus*, into the new *Notopappus* genus, comprising five species. This genus was proposed as differing from *Grindelia* by having a pappus with 20–60 persistent, unequal bristles united at the base to form a ring and cypselae with two narrow lateral ridges or wings.

According to previous taxonomic interpretations, *Notopappus* was established [15] based on species previously placed in *Haplopappus* Cass. sect. *Latiseta* Grau. These taxa had previously been transferred to *Grindelia* [1,28]. Accordingly, Klingenberg [15] segregated this genus, stating that these species did not conform to the diagnostic characters of either *Grindelia* or *Haplopappus*, and consequently elevated them back to species rank under a newly defined genus without phylogenetic backing.

Based on the morphological evidence obtained in the present study, *Grindelia mutabilis*, as described here, exhibits a combination of traits that challenges the current circumscription of *Notopappus* as distinct from *Grindelia* because it combines characters that have been assumed [15] to distinguish the two genera. The species shows a small stature and ovate, winged cypselae, characters traditionally attributed to *Notopappus*. However, it lacks the high number of pappus elements and the basal ring uniting them, features previously reported as diagnostic for *Notopappus* and commonly observed in *Grindelia*.

These inconsistencies have also been highlighted in previous taxonomic studies. In fact, they reflect broader taxonomic issues previously reported for the recognition of the *Notopappus* genus [17] when *N. pectinatus* (Phil.) Klingenb. was synonymized with *G. anethifolia* (Phil.) Adr.Bartoli & Tortosa, pointing out that the characters used to separate *Notopappus* from *Grindelia* were inconsistent. The number of pappus bristles, proposed as a distinguishing feature for *Notopappus* (20–60), was unreliable [14,15], as some *Grindelia* species have more bristles than the maximum attributed to the genus (2–10), suggesting a gradient rather than a definitive character for genera delimitation. This is further supported by the observation of other Brazilian species of *Grindelia*, such as *G. gaucha*, which has 13–17 pappus elements, and *G. buphthalmoides*., which has 21–24 pappus elements.

Previous studies also noted pappus persistence as inconsistent [17], as in some species, the bristles are initially united at the base by a ring but become free and deciduous at maturity, unlike the persistent bristles attributed to *Notopappus*. Additionally, the use of cypsela shape as a distinguishing feature was found to be variable within *Grindelia* [14].

Published molecular phylogenetic reconstructions of *Grindelia* [8] based on nuclear ribosomal and chloroplast DNA do not support the recognition of *Notopappus* as a distinct genus. The South American species previously transferred to *Notopappus* [15]—namely, *Grindelia anethifolia* and *G. prunelloides* (Poepp. ex Less.) Adr.Bartoli & Tortosa—are not sister species and do not emerge as a clade in relation to the remaining South American *Grindelia*. Instead, *G. anethifolia* forms a clade with *G. chiloensis*, *G. coronensis*, and *G. mendocina* Adr.Bartoli & Tortosa, while *G. prunelloides* emerges with *G. pygmaea* Cabrera, each within a distinct clade of the two major South American clades of *Grindelia* [8]. This pattern demonstrates that *Notopappus* is polyphyletic, as its species share closer phylogenetic affinities with other South American taxa of *Grindelia* than with each other and do not constitute an isolated lineage that would justify generic separation. Moreover, accepting *Notopappus* would render *Grindelia* non-monophyletic.

To date, no formal taxonomic treatment has been published to reinforce the synonymization of *Notopappus* in *Grindelia*, and some specialized literature [16] and reference databases [32] still accept *Notopappus*. Considering the combination of morphological evidence obtained in the present study, previous taxonomic assessments highlighting morphological inconsistencies [14,15,17], and previously published phylogenetic results [8], the sampled *Notopappus* species emerged within *Grindelia*, embedded within the clade of South American species. In addition to the prior synonymization of *Notopappus pectinatus*, which is also the type species of *Notopappus* [17], and the existence of several morphologically intermediate species between the two genera discussed here, including the new species described herein, we therefore propose the synonymization of *Notopappus* under *Grindelia* and the re-establishment of its species within *Grindelia*.

### 3.4. Taxonomic and Typification Novelties

 ***Grindelia*** Willd., Mag. Neuesten Entdeck. Ges. Naturk. Deutsch. Naturf. 1: 259 (1807). **Type** (Lectotype designated by [4], p. 474): *Grindelia inuloides* Willd. =*Notopappus* Klingenb., Biblioth. Bot. 157: 69 (2007), **syn. nov.** Type (lectotype designated by [15], p. 100): *Haplopappus pectinatus* Phil. = *Notopappus pectinatus* (Phil.) Klingenb., Biblioth. Bot. 157: 92 (2007). Lectotype: Chile. Philippi s.n. (SGO44156 digital image!) (Figure S1); isolectotype: SGO57484. ***Grindelia andina*** Phil., Linnaea 33(2): 137 (1864). =*Chrysophithalmum andinum* (Phil.) Phil., Linnaea 29: 9 (1858). =*Notopappus andinus* (Phil.) Klingenb., Biblioth. Bot. 157: 96 (2007), **syn. nov**. **Type** (lectotype designated by [15], p. 96): CHILE. Andes de Linares, Jan–Feb, P. Germain s.n. (SGO65019 digital image!) (Figure S2). ***Grindelia prunelloides*** (Poepp.) A.Bartoli & Tortosa, Hickenia 3(3): 33 (1999). =*Diplopappus prunelloides* Poepp. ex Less., Linnaea 6: 111 (1831). **Type:** CHILE. a planitie areosa ad pedem mont. igniv. Antuc. circum la Cueva. Poeppig,E.F. Diar. 917 Coll. pl. Chil. III 204. (Holotype: HAL0111018 digital image!, Isotype: GH00139463 digital image!). =*Haplopappus prunelloides* (Poepp. ex Less.) DC., Prodr. [A. P. de Candolle] 5: 346 (1836). =*Aster prunelloides* (Less.) Kuntze, Revis. Gen. Pl. 1: 318 (1891). =*Notopappus prunelloides* (Poepp. ex Less.) Klingenb., Biblioth. Bot. 157: 100 (2007), **syn. nov.** **Type** (lectotype designated by [15], p. 100): CHILE. Cresc. in Chile a planite arenosa ad pedem mont. Igniv. Antuc., circum la Cueva, Febre. Lect., Diar. 917. Poeppig 204 (Lectotype: G00457735 digital image!; isolectotypes: BM, M0029623 digital image!, P, GH00139463 digital image!). =*Haplopappus ameghinoi* Speg., Anales Soc. Ci. Argent. 48: 180 (1899). =*Notopappus ameghinoi* (Speg.) Klingenb., Biblioth. Bot. 157: 89 (2007), **syn. nov.** **Type** (lectotype designated here): ARGENTINA. Santa Cruz, Chonkenkaik, Río Chico, en el fondo de lagunas secas y guadales, I-1897, C. Ameghino 79 (Lectotype: LP000079! (Figure S3); Syntypes: C. Ameghino 131 (LP000078! Figure S4), C. Ameghino s.n. (LP000080!) (Figure S5).*Typification note:* LP000079 is designated here as the lectotype because it preserves the most complete set of diagnostic features, including root structure, leaves, and capitula. In contrast, the other syntypes (LP000078 and LP000080) are more fragmented and do not, individually, exhibit all the diagnostic characters of the species.  =*Haplopappus illini* Speg., Anales Soc. Ci. Argent. 48: 181 (1899). Type: ARGENTINA. Inter Choique-laüen y lago Musters, enero 1899, N. Illín s.n. (Holotype: LPS100 en LP000082! (Figure S6); Isotype: LP000081! ex LPS-100) (Figure S7). =*Haplopappus prunelloides* var. *sphaerocephalus* Reiche, Anales Univ. Chile 109: 50 (1901). Type: CHILE. Cordillera de Linares, R. A. Philippi s.n. (Holotype: SGO57490, digital image!) (Figure S8). =*Haplopappus mustersi* Speg., Anales Soc. Ci. Argent. 48: 183 (1899). =*Haplopappus prunelloides* var. *mustersii* (Speg.) Cabrera in M.N.Correa, Fl. Patagonica 7: 56 (1971). Type: ARGENTINA. Chubut, depti. Sarminto, entre Choique-lahuen y lago Musters, enero 1899, N. Illin 86 (Holotype: LP000084! (Figure S9); Isotype: LP000085!) (Figure S10). =*Haplopappus bellidifolius* Phil., Anales Univ. Chile 87: 593 (1894). Type: CHILE. Guaieltué, Febr. 1887, C. Rahner s.n. (Holotype: SGO57441, digital image!) (Figure S11). =*Haplopappus prunelloides* var. *lanatus* Cabrera in M.N.Correa, Fl. Patagonica 7: 56 (1971). Type: ARGENTINA. Neuquén, Ea. Llaueuco, ruta 235, S. Schajovskoy 302 (Holotype: LP000038!) (Figure S12). =*Haplopappus bellidifolius* var. *brachylepis* Reiche, Anales Univ. Chile 109: 51 (1901). =*Haplopappus brachylepis* (Reiche) H.M.Hall, Univ. Calif. Publ. Bot. 7: 273 (1919). Type: CHILE. Araucanía?, Philippi s.n. (Holotype: SGO57443, digital image!) (Figure S13). ***Grindelia prunelloides*** var. ***discoides*** Tortosa & Adr.Bartoli, Bol. Soc. Argent. Bot. 36(1-2): 142 (2001). Type: ARGENTINA. Neuquén, Dpto. Minas: Sierra de Cochicó, cumbre, 29 Ene 1970, O. Boelcke 14,077 (Holotype: BAA00001242!). =*Pyrrocoma aurea* Phil., Linnaea 28: 733 (1858). =*Aster chryseus* Kuntze, Revis. Gen. Pl. 1: 315 (1891). =*Haplopappus chrysocephalus* Reiche, Anales Univ. Chile 109: 37 (1901). =*Haplopappus chryseus* (Kuntze) Cabrera, Fl. Patagónica, Colecc. Ci. Inst. Nac. Tecnol. Agro-pec. 8(7): 54 (1971). =*Notopappus chryseus* (Kuntze) Klingenb., Biblioth. Bot. 157: 96 (2007), **syn. nov.** Type (lectotype designated by [15], p. 96): CHILE. Cordillera de Linares, Enero 1856, P. Germain s.n. (Lectotype: SGO44371, digital image! (Figure S14); Isolectotype: SGO57435, digital image!) (Figure S15).

## 4. Materials and Methods

Literature on South American *Grindelia* [1,2,3,4,5,6,7,28,29,30,31,33,34,35,36,37,38,39,40,41] and *Notopappus* [15] was consulted, including protologues, floras and taxonomic revisions. To support the current new species description and newly proposed synonyms, the following Argentine, Brazilian and Uruguayan herbaria were consulted: BAA, BHCB, CEN, ECT, FLOR, HAS, HDCF, HUCS, HURG, ICN, LP, MBM, MPUC, MVFA, MVJB, MVM, PACA, PEL, R, RB, SI, SP, and SPF (acronyms according to Thiers [42], continuously updated). All herbaria were consulted either in person or through physical specimen loans. Additionally, the following herbaria were consulted through online platforms or by requesting images of specific specimens: BM, F, GH, M, P, and SGO. Type materials were examined either personally, via photographs available on JSTOR [43], or through direct image requests. Scientific names and authorities were verified following the IPNI (International Plant Names Index) [44]. Fieldwork was conducted in Espinilho State Park in December 2024 (license number: 00036/2024) to document the new species in its habitat and gather data on population status and ecological preferences. However, no new samples were collected during this time due to few living specimens being found. An additional search of new populations was performed in the surrounding areas within and outside of the state park, and no additional specimens were found.

Morphological description was based on vegetative and reproductive material from herbarium specimens and photographic records of living plants. Measurements were taken using a Leica M165 C, Wetzlar, Germany, stereomicroscope equipped with a digital caliper. Vegetative structures were analyzed from dried material, while reproductive structures were examined after rehydration by immersing them in warm water for one minute. The indumentum and textures were observed through photographic analysis of both living and dried material. The general terminology for morphological features, measurements, and color descriptions follows [45,46], while the specialized terminology for Compositae follows [47,48]. *Grindelia* leaves, bracts and bracteoles were described as defined by [6].

Based on photographs of living specimens and type collections, the new species was illustrated using watercolor to represent habit, leaves, bracts, bracteoles capitula, flowers and cypselae. The illustration was prepared on Arches^®^, Arches, France, 100% cotton, acid-free, watercolor paper using Caran D’Ache^®^, Geneva, Switzerland, colored pencils. The illustration was post edited, and scale bars were added using Adobe Photoshop 2025^®^, San Jose, CA, USA.

The geographic distribution map was prepared using Quantum GIS version 3.40.3, Switzerland [49]. The preliminary conservation status assessment followed the IUCN (International Union for Conservation of Nature) Red List Categories and Criteria [23], with an attempt to apply all criteria (A–E), depending on data availability, using GeoCAT (Geospatial Conservation Assessment Tool, Richmond, UK) [24] to calculate the Extent of Occurrence (EOO) and Area of Occupancy (AOO, 2 × 2 km grid) under criterion B.

## 5. Conclusions

*Grindelia mutabilis* is a distinct new species proposed here and characterized by the unique combination of a 0.2–0.3 m tall cespitose rosette habit; linear to linear–oblanceolate leaves; light-yellow to pastel-salmon ray corollas; three-winged ray floret cypselae bearing a pappus of two to four elements and two-winged disc-floret cypselae bearing a pappus of two elements, also providing further evidence supporting the synonymization of *Notopappus*, reinforcing conclusions previously suggested by both taxonomic and molecular phylogenetic studies. This study represents a step forward in clarifying the taxonomy of the genus, recognizes a microendemic endangered species and contributes to the organization and stabilization of nomenclature and taxonomic relationships among South American taxa within the Machaerantherinae subtribe from the Astereae tribe.

## Data Availability

The original contributions presented in this study are included in the article/Supplementary Material. Further inquiries can be directed to the corresponding author.

## Supplementary Materials

The following supporting information can be downloaded at: https://www.mdpi.com/article/10.3390/plants15050760/s1, Figure S1–S15: photographs of herbarium specimens that are not available online in high quality.

## Abbreviations

The following abbreviations are used in this manuscript:

PAFTOL	Plant and Fungal Tree of Life
WWF	World Wide Fund for Nature
IUCN	International Union for Conservation of Nature
EOO	Extent of Occurrence
AOO	Area of Occupancy
GeoCAT	Geospatial Conservation Assessment Tool
IPNI	International Plant Names Index
