# Supplementary material for: Grindelia mutabilis (Asteraceae: Astereae), a New South American Species and a Link for Synonymizing Notopappus
Source: Plants (Basel). 2026 Mar 1;15(5):760. doi: 10.3390/plants15050760 (PMC12987375; doi:10.3390/plants15050760)
Supplement: Supplementary file 1 [file plants-15-00760-s001.zip › plants-4116991-supplementary.pdf]

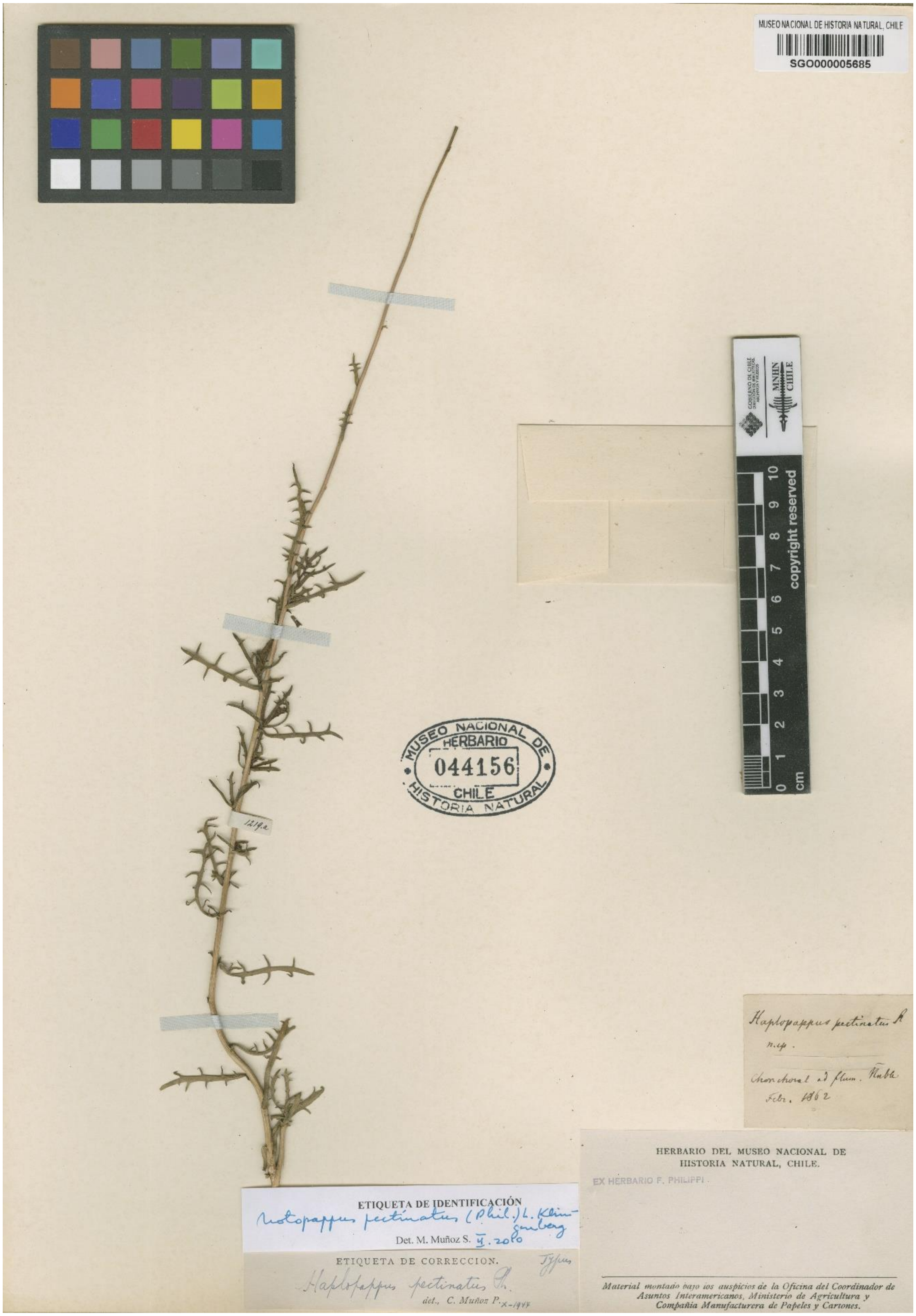

Figure S1. SGO44156, lectotype of *Notopappus pectinatus* (Phil.) Klingenb.

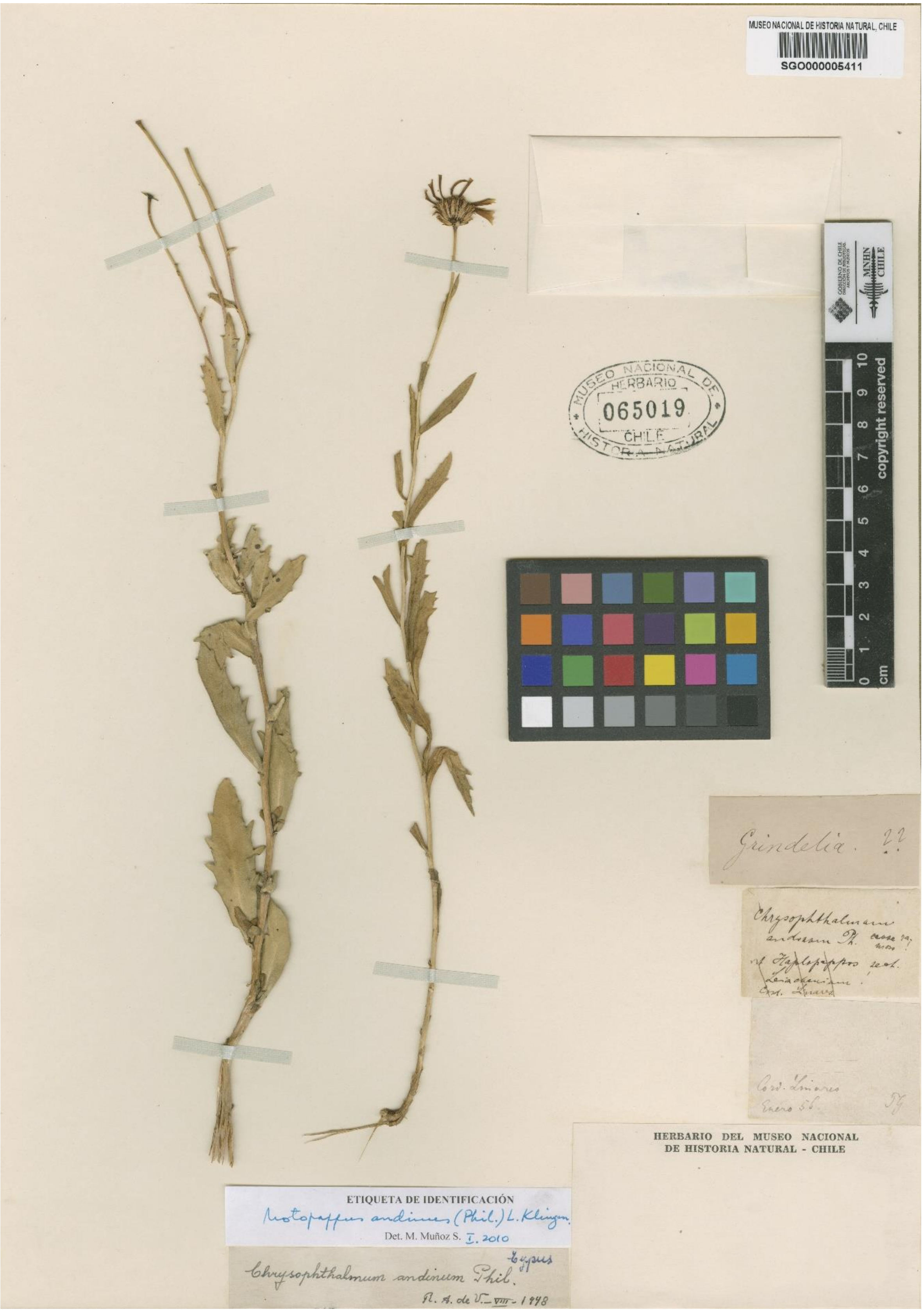

Figure S2. SGO65019, lectotype of *Notopappus andinus* (Phil.) Klingenb

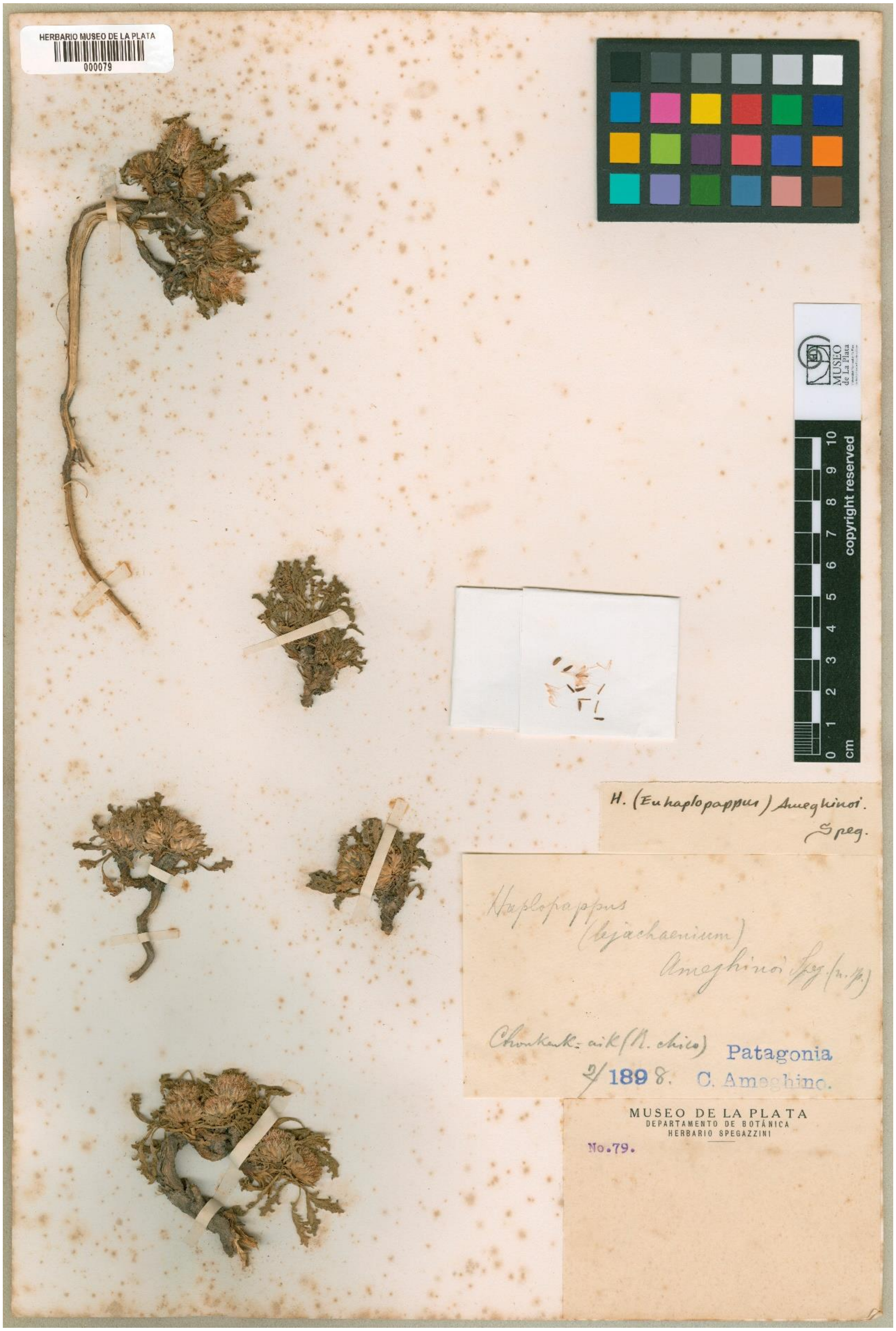

Figure S3. LP000079, lectotype of Notopappus ameghinoi (Speg.) Klingenb.

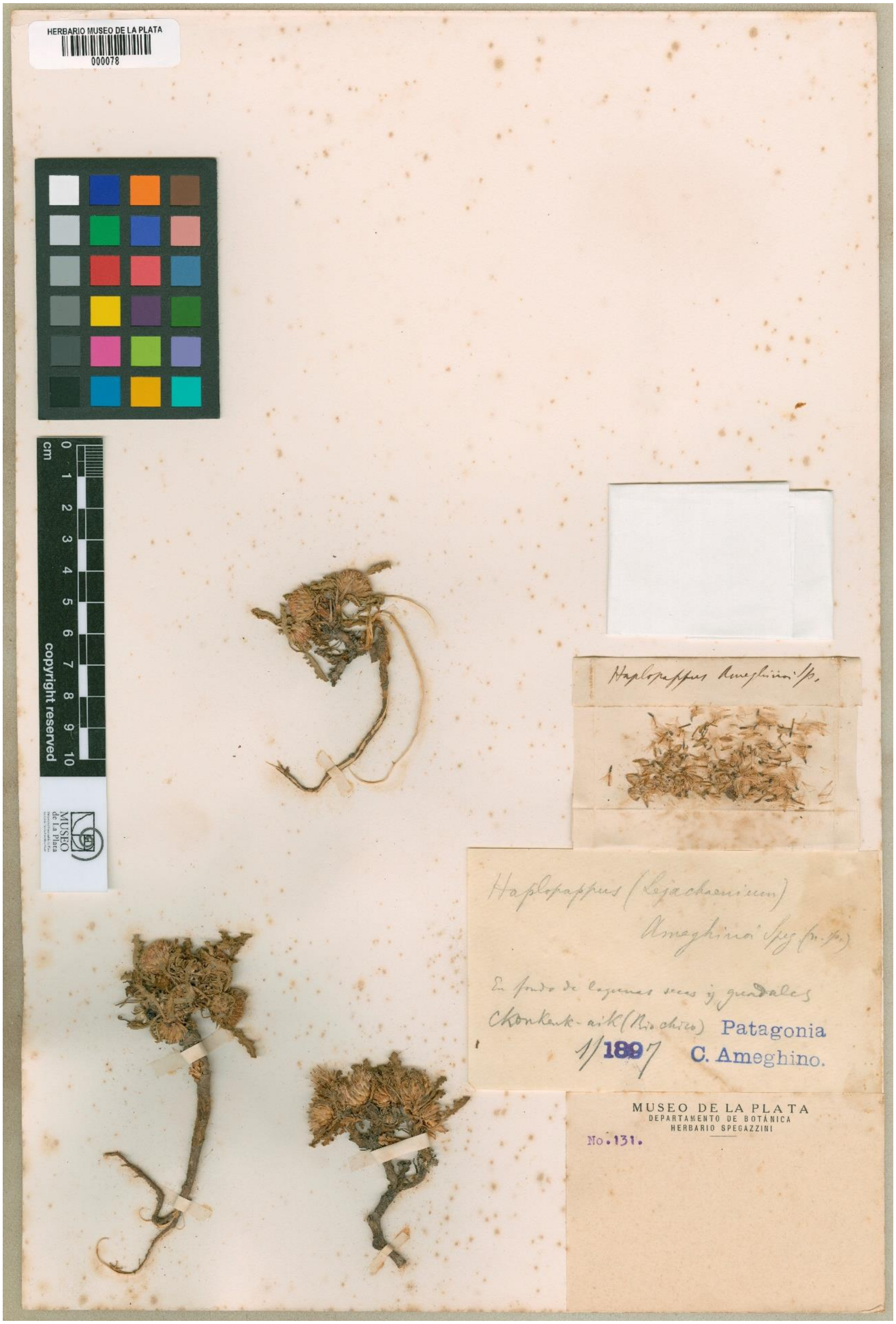

Figure S4. LP000078, syntype of Notopappus ameghinoi (Speg.) Klingenb.

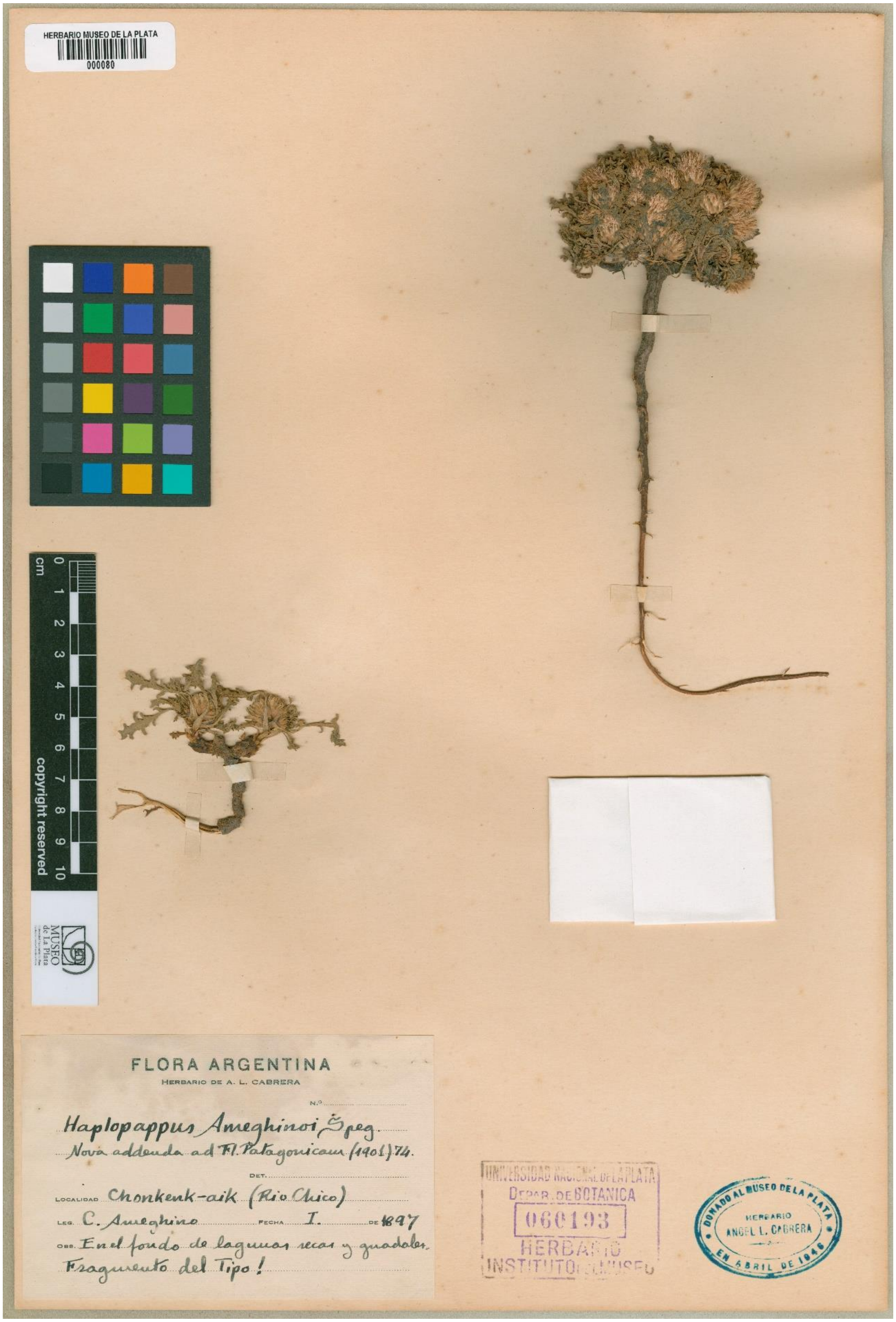

Figure S5. LP000080, syntype of Notopappus ameghinoi (Speg.) Klingenb.

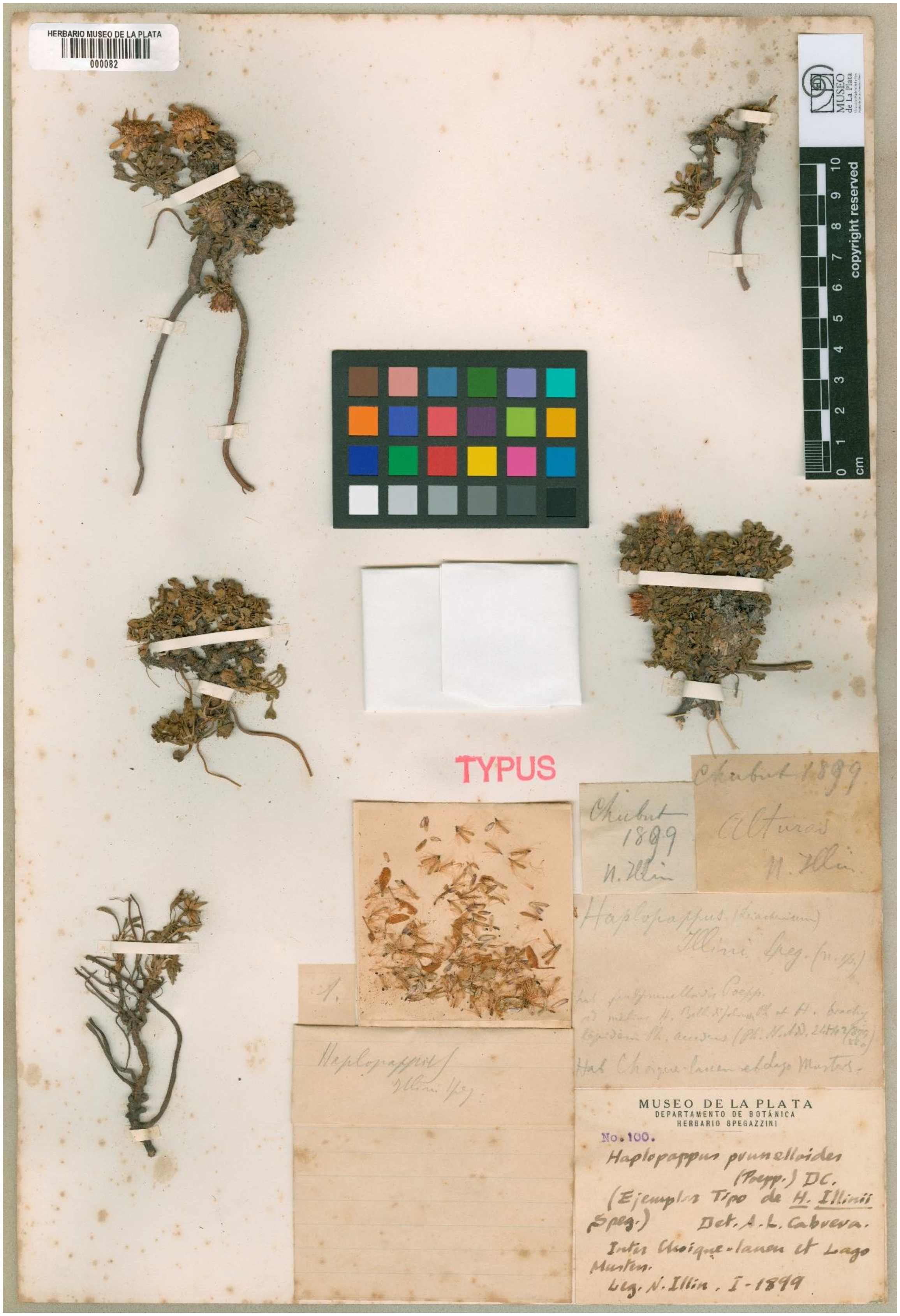

Figure S6. LP000082, holotype of *Haplopappus illini* Speg.

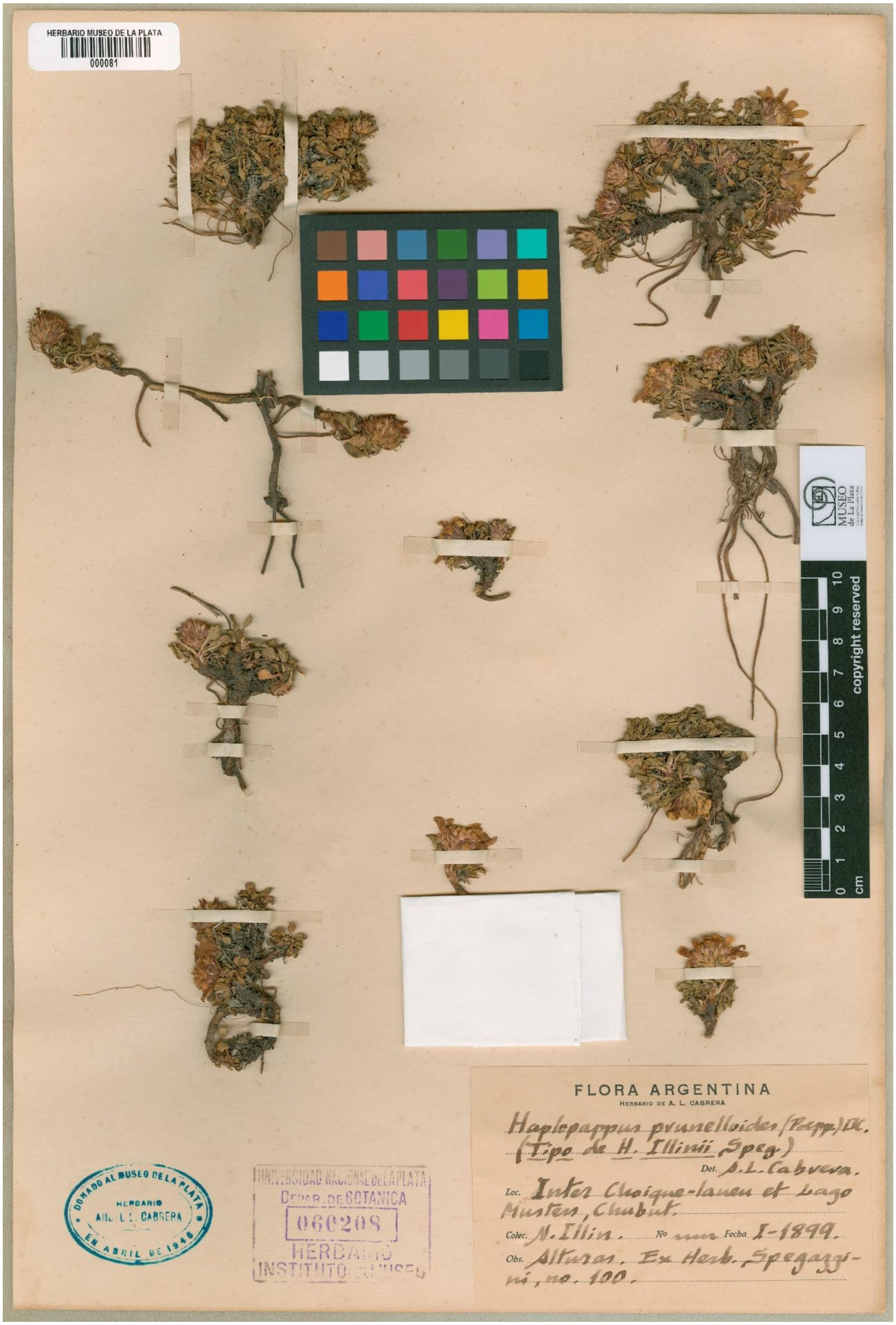

Figure S7. LP000081, isotype of *Haplopappus illini* Speg.

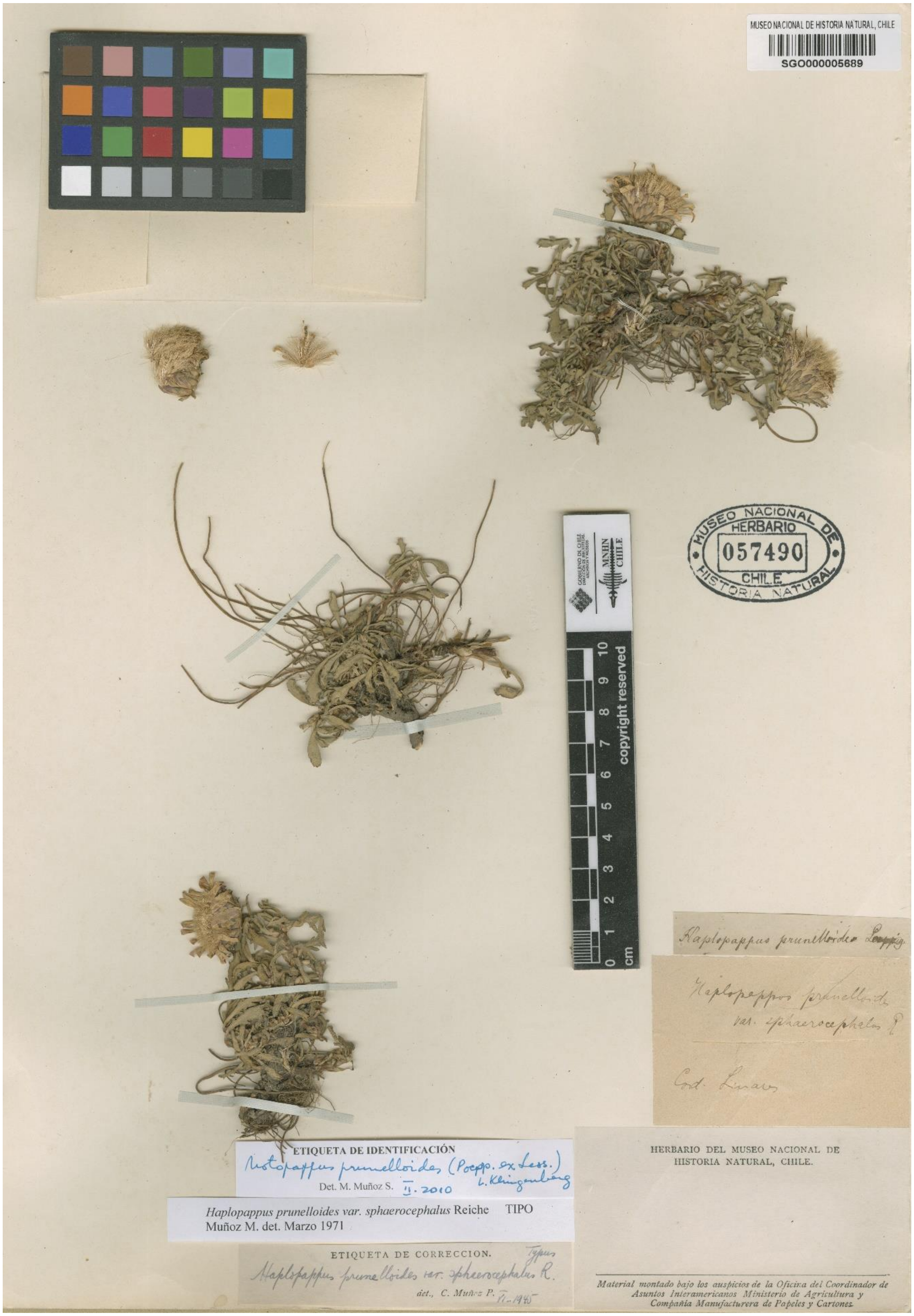

Figure S8. SGO57490, holotype of *Haplopappus prunelloides* var. *sphaerocephalus* Reiche

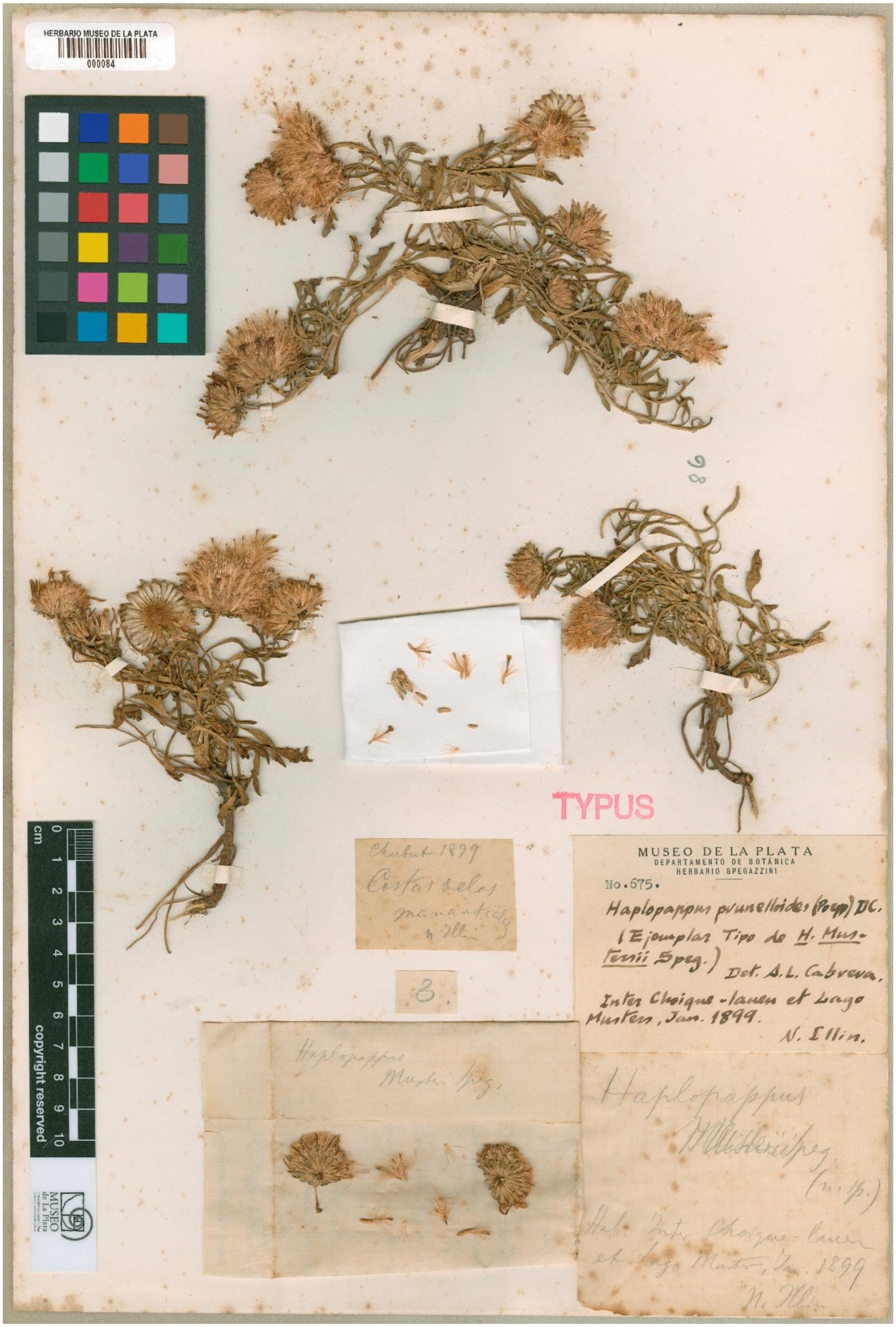

Figure S9. LP000084, holotype of *Haplopappu prunelloides* var. *mustersii* (Speg.) Cabrera

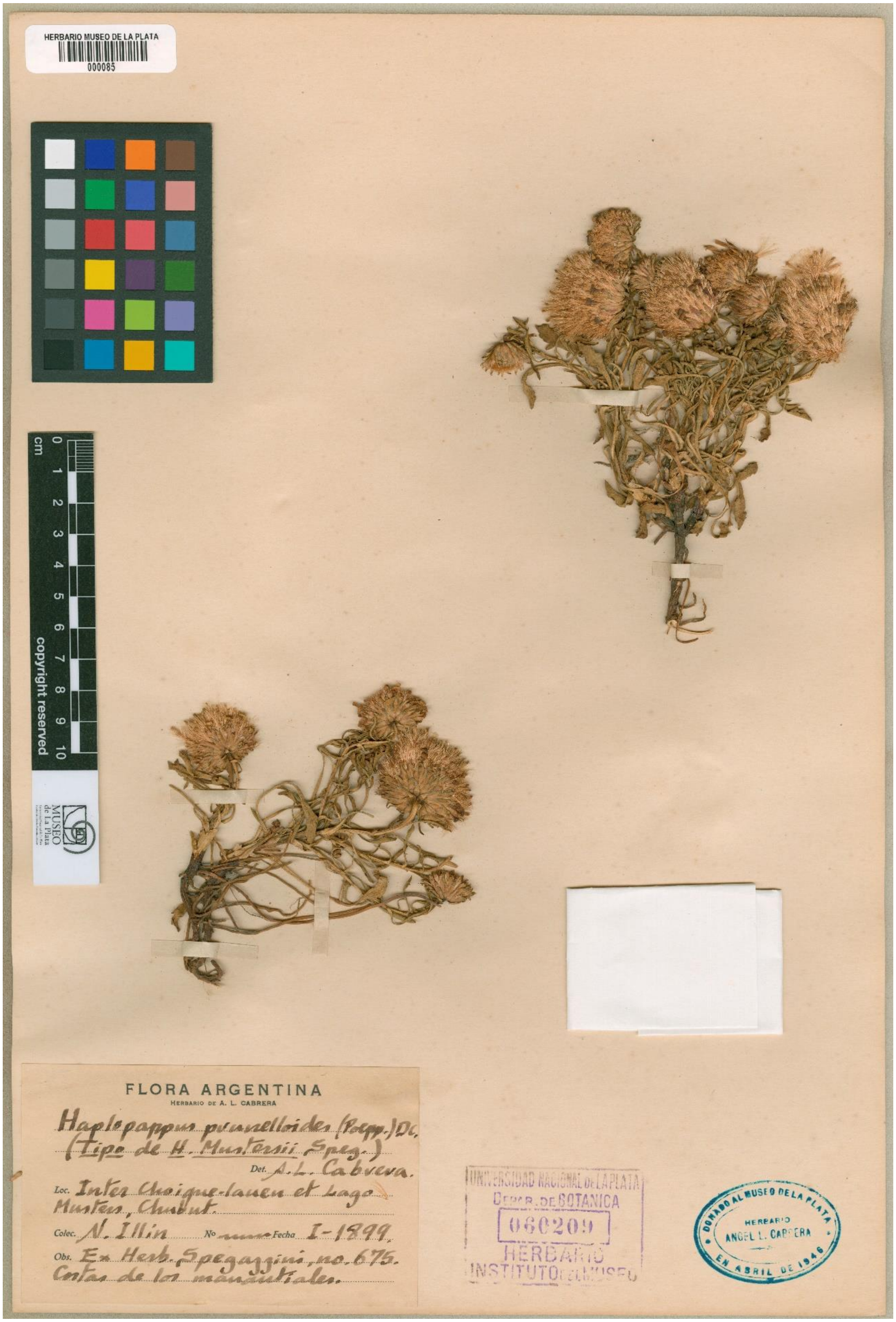

Figure S10. LP000085, isotype of *Haplopappu prunelloides* var. *mustersii* (Speg.) Cabrera

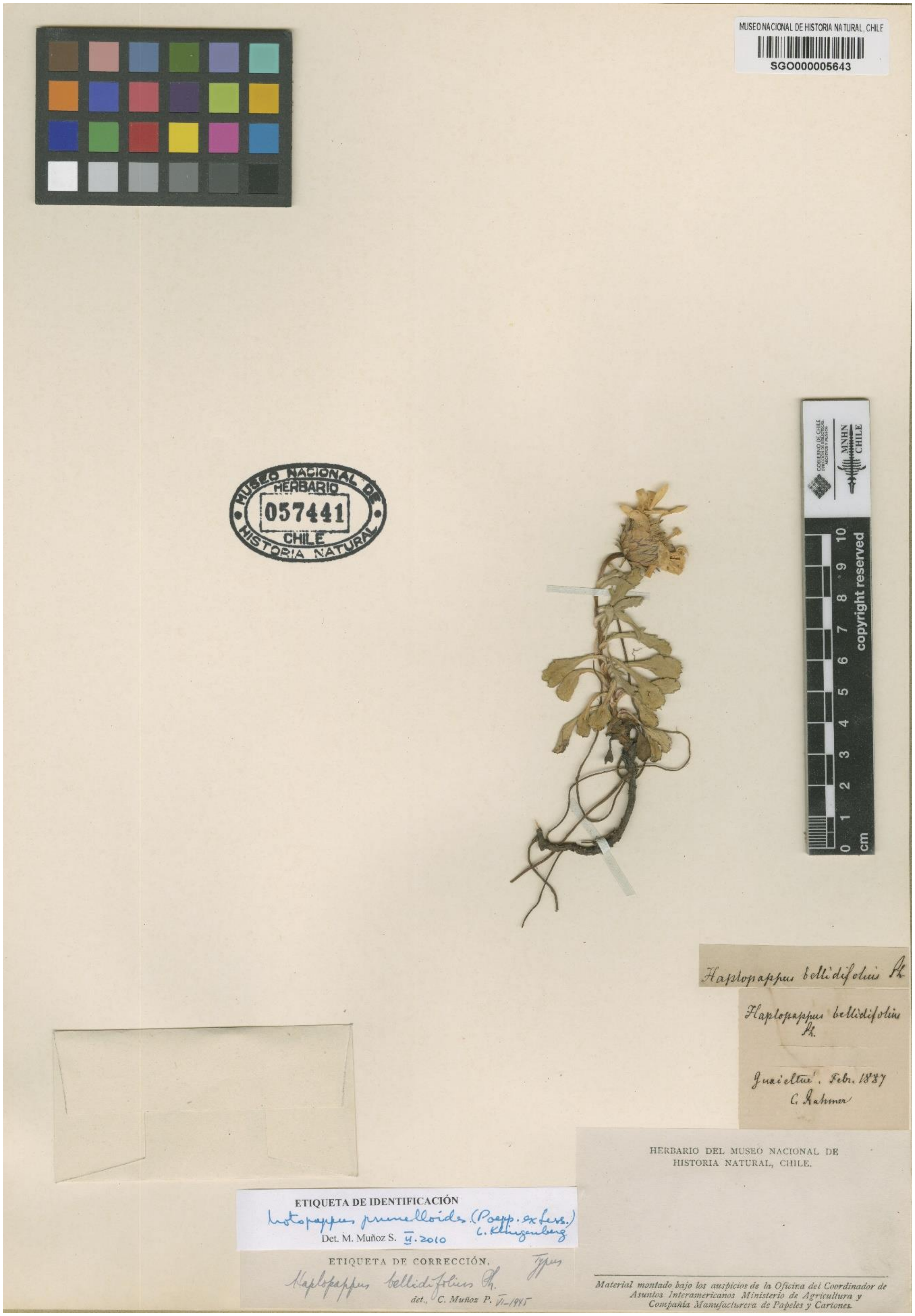

Figure S11. SGO57441, holotype of *Haplopappus bellidifolius* Phil.

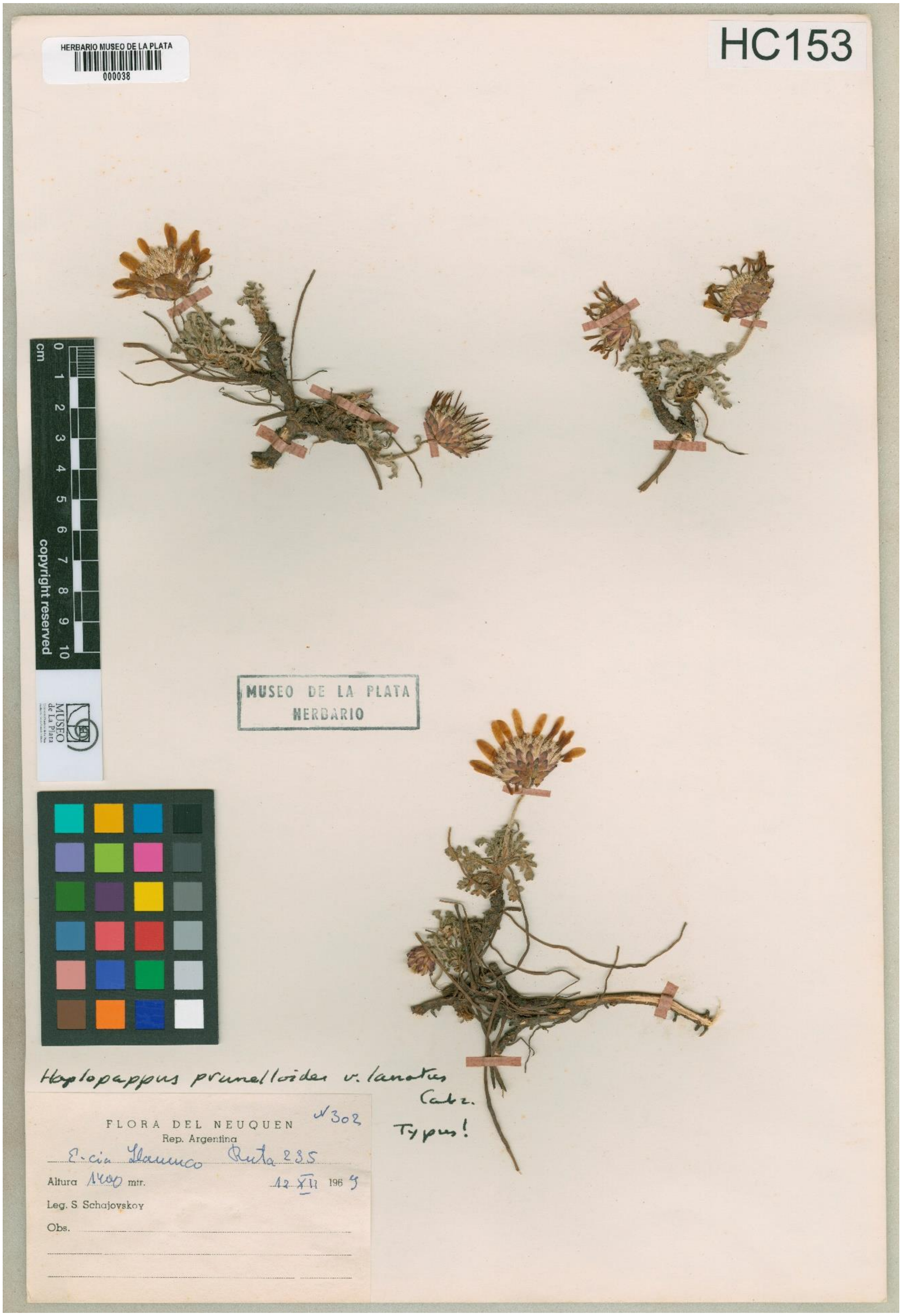

Figure S12. LP000038, holotype of *Haplopappus prunelloides* var. *lanatus* Cabrera

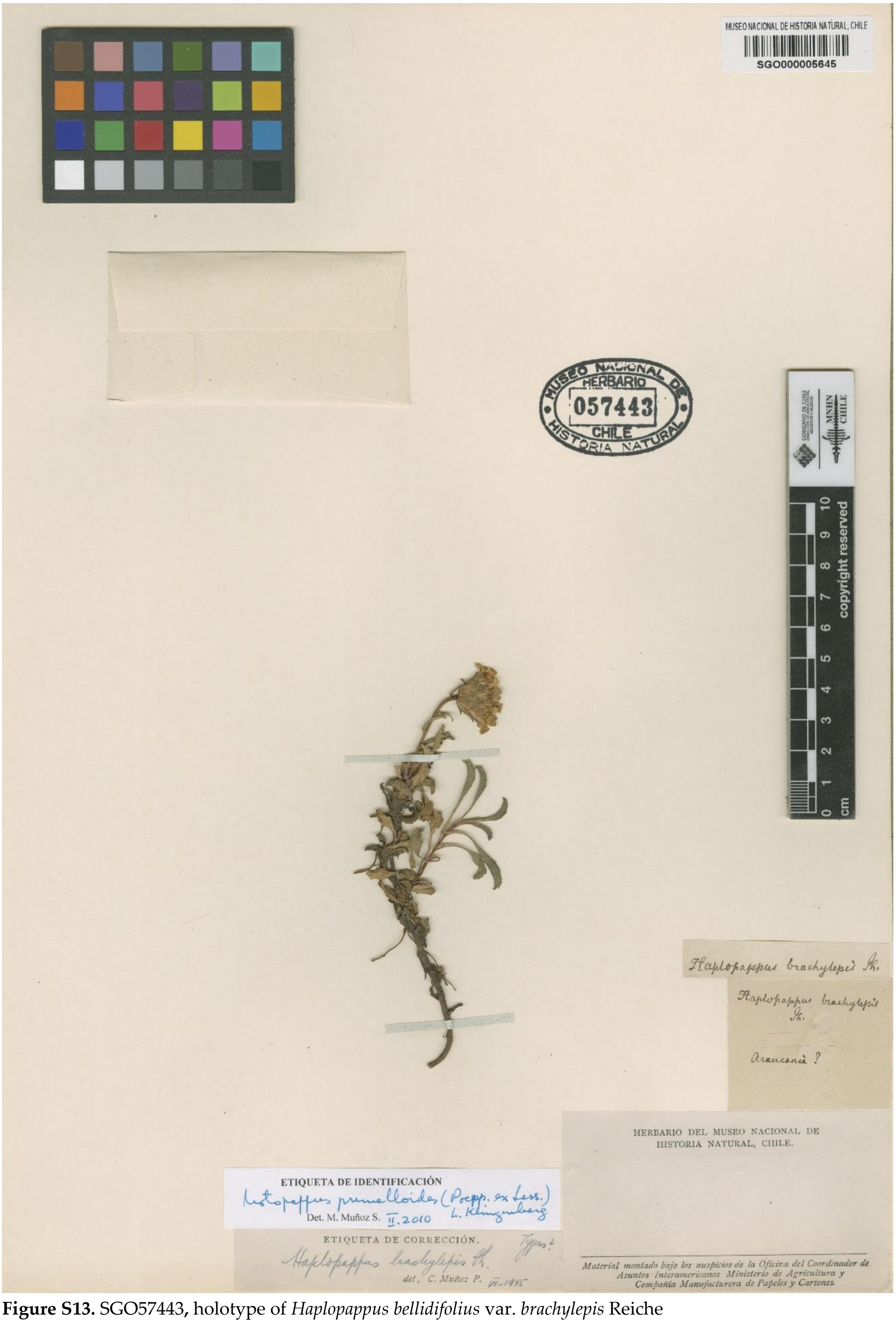

Figure S13. SGO57443, holotype of *Haplopappus bellidifolius* var. *brachylepis* Reiche

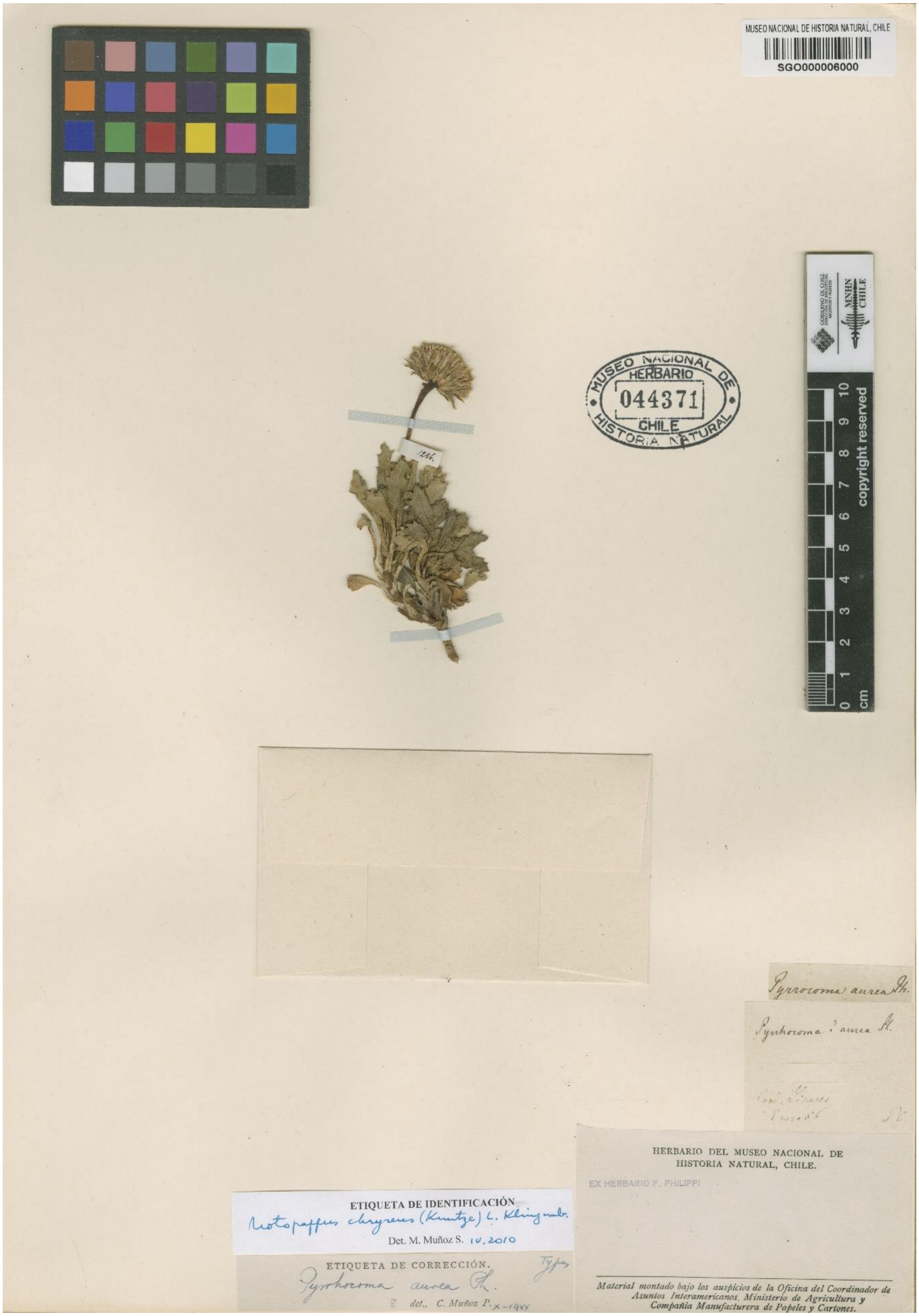

Figure S14. SGO44371, lectotype of *Notopappus chryseus* (Kuntze) Klingenb.

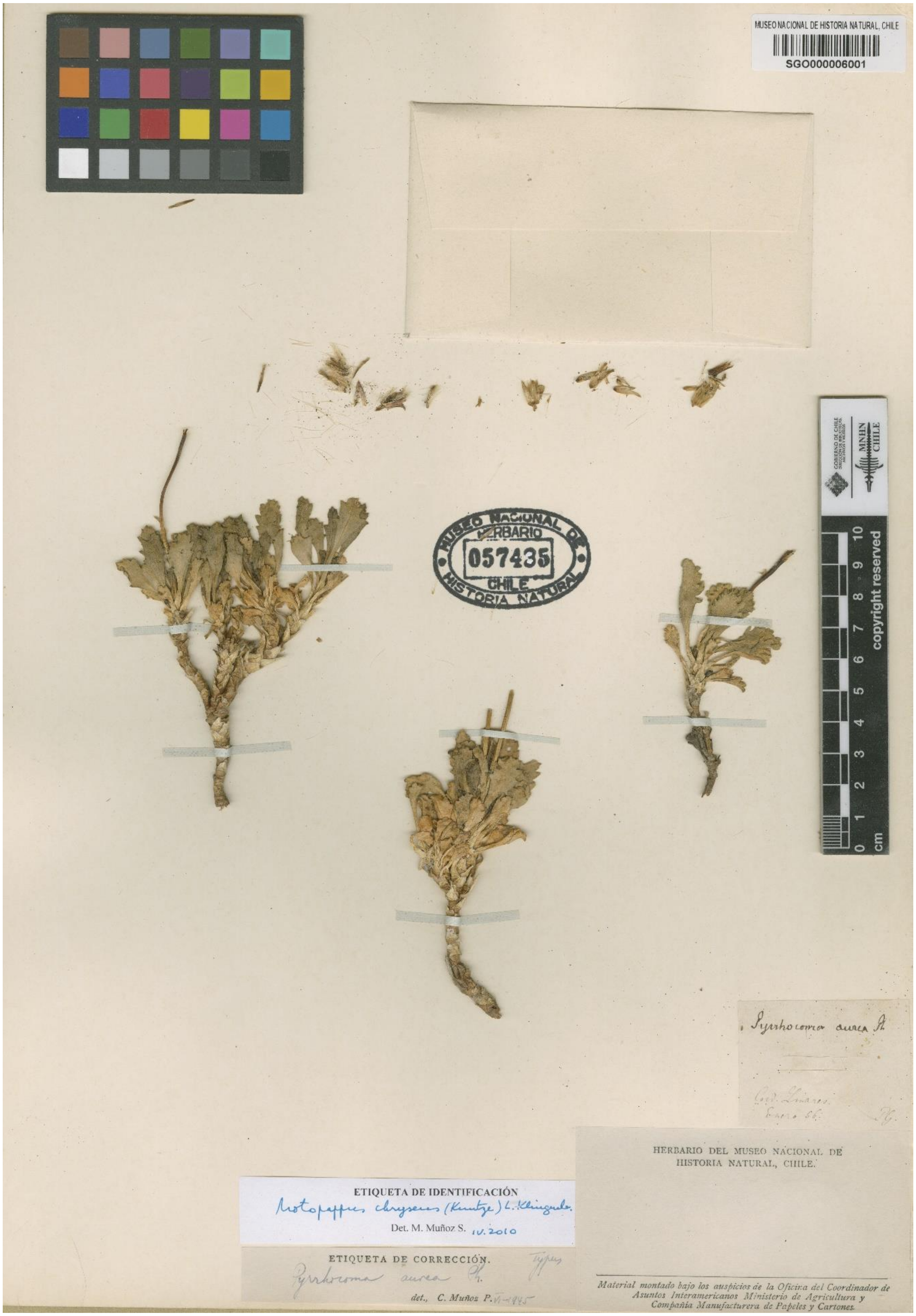

Figure S15. SGO57435, isolectotype of *Notopappus chryseus* (Kuntze) Klingenb.
